# Antiviral Strategies of Chinese Herbal Medicine Against PRRSV Infection

**DOI:** 10.3389/fmicb.2020.01756

**Published:** 2020-07-28

**Authors:** God’spower Bello-Onaghise, Gang Wang, Xiao Han, Eliphaz Nsabimana, Wenqiang Cui, Fei Yu, Yuefeng Zhang, Linguang Wang, Zhengze Li, Xuehui Cai, Yanhua Li

**Affiliations:** ^1^Heilongjiang Key Laboratory for Animal Disease Control and Pharmaceutical Development, College of Veterinary Medicine, Northeast Agricultural University, Harbin, China; ^2^Department of Animal Science, Faculty of Agriculture, University of Benin, Benin City, Nigeria; ^3^State Key Laboratory of Veterinary Biotechnology, Harbin Veterinary Research Institute, Chinese Academy of Agricultural Sciences, Harbin, China; ^4^Department of Animal and Veterinary Science, Chengdu Agricultural College, Chengdu, China; ^5^Department of Chinese Medicine, Jiangxi University of Traditional Chinese Medicine, Nanchang, China

**Keywords:** Traditional Chinese Medicine, bioactive compounds, cytokines, immunity, porcine reproductive and respiratory syndrome virus, life cycle, antiviral activity

## Abstract

Bioactive compounds from Traditional Chinese Medicines (TCMs) are gradually becoming an effective alternative in the control of porcine reproductive and respiratory syndrome virus (PRRSV) because most of the commercially available PRRSV vaccines cannot provide full protection against the genetically diverse strains isolated from farms. Besides, the incomplete attenuation procedure involved in the production of modified live vaccines (MLV) may cause them to revert to the more virulence forms. TCMs have shown some promising potentials in bridging this gap. Several investigations have revealed that herbal extracts from TCMs contain molecules with significant antiviral activities against the various stages of the life cycle of PRRSV, and they do this through different mechanisms. They either block PRRSV attachment and entry into cells or inhibits the replication of viral RNA or viral particles assembly and release or act as immunomodulators and pathogenic pathway inhibitors through cytokines regulations. Here, we summarized the various antiviral strategies employed by some TCMs against the different stages of the life cycle of PRRSV under two major classes, including direct-acting antivirals (DAAs) and indirect-acting antivirals (IAAs). We highlighted their mechanisms of action. In conclusion, we recommended that in making plans for the use of TCMs to control PRRSV, the pathway forward must be built on a real understanding of the mechanisms by which bioactive compounds exert their effects. This will provide a template that will guide the focus of collaborative studies among researchers in the areas of bioinformatics, chemistry, and proteomics. Furthermore, available data and procedures to support the efficacy, safety, and quality control levels of TCMs should be well documented without any breach of data integrity and good manufacturing practices.

## Introduction

The pathogen; porcine reproductive and respiratory syndrome virus (PRRSV), as well as other members, for example, EAV, LDV, and SHFV, belong to the large family of Arteriviridae, which is made up of enveloped, positive-strand RNA viruses (Lunney et al., 2016). PRRSV exists as two distinct virus species, i.e., PRRSV-1 (prototype Lelystad)_ and PRRSV-2 (prototype VR-2332), and the genome is about 15 kb in length, and they share ∼60% nucleotide identity (2016)^1^ (Meng et al., 1995). PRRSV is the pathogen of Porcine reproductive and respiratory syndrome (PRRS). It is the leading cause of pregnancy wastages and piglet mortality in swine herds. The current commercially available inactivated and MLV cannot provide full protection against the genetically diverse strains in the field in most cases (Meng, 2000), and MLV have the potential of reverting to more virulent forms, which cause more infection than prevention (Kimman, 1992; Lei and Yang, 2010). Besides, the humoral and cell-mediated immune (CMI) responses elicited by PRRSV-MLVs is delayed and relatively weak (Diaz et al., 2006; Zuckermann et al., 2007). For example, it takes a delayed period of 28–30 days for the PRRSV-specific neutralizing antibodies (NA) responsible for the clearance of PRRSV from the pigs to appear after vaccination with a relatively low titer (usually between 8 and 32) (Darwich et al., 2010). T cell response to PRRSV-MLVs is extremely delayed compared with T cell response to other RNA viruses. That of PRRSV appears in about 7–14 days and peaks between 220 and 224 days after vaccination, while that of pseudorabies virus (PRV)is observed within 7 days of vaccination and peaks approximately within 28–30 days after vaccination (Meier et al., 2003). Furthermore, PRRSV-MLV vaccinated pigs can develop viremia for up to 30 days after vaccination, increasing the risk of vaccine virus transmission to naive animals (Charerntantanakul, 2012; Wang et al., 2013c). The induction of antibody-dependent enhancement (ADE) of infection has also be reported in herds vaccinated with MLV (Jiang et al., 2003; Zhou et al., 2004). Thus, the need for an alternative therapy that is potent, inexpensive, efficient and effective against PRRSV is critically urgent. PRRSV infection is highly restricted to cells *in vitro* and *in vivo*. PRRSV infection in the host cells referred to the four stages: entry (attachment and internalization), uncoating, nucleic acid or protein synthesis, assembly, and viral release (Klasse et al., 1998). Viral attachment to putative receptors expressed at the cell surface is the first phase in the process of viral entry into susceptible cells. Molecules or factors including HS, vimentin, cluster of differentiation 151 (CD151), CD163, sialoadhesin (CD169, Sn), DC-SIGN (CD209), and non-muscle myosin heavy chain 9 (MHC II-A or MYH9) are among the several proteins described as putative receptors for PRRSV (Gao et al., 2016; Lunney et al., 2016).

### PRRSV Infection

**FIGURE 1:**
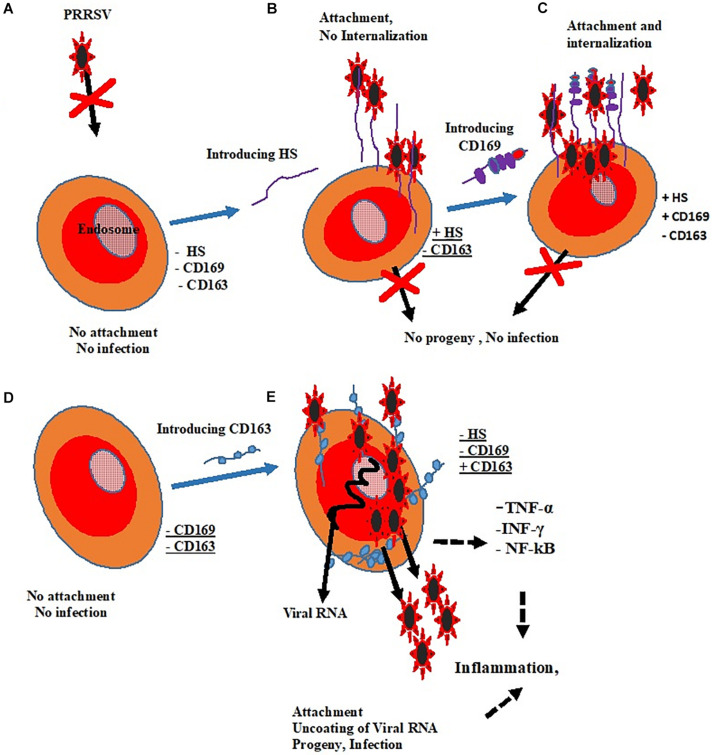
Roles of putative receptors in PRRSV infectivity. **(A)** Cell without receptors shows no susceptibility. **(B)** The expression of HS could only result in viral attachment. **(C)** CD169 promotes attachment and internalization without resultant infection. **(D)** There is neither attachment nor infection in the absence of CD169 and CD163. **(E)** The presence of CD163 alone changed non-permissive cell to permissive cell and results in positive infection. Key: Heparan Sulfate (HS) = 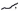 Sialoadhesin (CD169) = 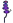 Cluster of Differentiation (CD163) = 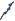.

Pigs are the natural host of PRRSV, and the virus infects a narrow range of cells, both *in vitro* and *in vivo* due to the presence of putative receptors in these cells. Porcine alveolar macrophages and blood monocytes are the only porcine cells known to be susceptible to PRRSV infection due to the abundant presence of putative receptors on the surfaces of these cells; however, some cells from the African green monkey kidney cell lines including CL2621, MA-104, and MARC-145 are used for research and propagation of PRRSV *in vitro* because they express varying levels of PRRSV receptors thereby supporting infection by the virus. These receptors play significant roles during PRRSV infection of cells since they can be involved in the virus binding, internalization or uncoating. Among these, CD163 is indispensable for positive infection (Gao et al., 2016) and SIGLEC1 or CD169 is not necessary for PRRSV infection (Prather et al., 2013), see Figure 1.

The initial primary defense response of animals to viral infection is the production of interferon-alpha (IFN –α), (a type 1 interferon), often followed by inflammatory cytokines such as tumor necrosis factor (TNF) and interleukin-1 (IL-1). However, with PRRSV infection, such response is weakened or significantly suppressed. Some authors have stated that the expression of IFN –α inhibits the replication of viruses, including PRRSV *in vitro* (Albina et al., 1998; Zhang et al., 2014; Brockmeier et al., 2017). The expression of inflammatory cytokine plays a very significant role in the organization of the host immune system against a variety of viral infection (Brockmeier et al., 2017). TNF, IL-1β, and IL-6 are essential activators of the nuclear transcription factor, NF-κB (Christman et al., 2000; Mori et al., 2011). The function of NF-κB during infection is to regulate the transcription of hundreds of genes, including antigen receptors, inflammatory and immunoregulatory cytokines, adhesion molecules, inhibitors of apoptosis, acute phase proteins, and innate effector molecules; for this reason, it was stated that the mild or subclinical respiratory abnormalities involved in PRRSV infection might be due to the lack of activation of NF-κB. However, when there is a lack of an acute inflammatory response and weak innate antiviral activity, the resultant effect may also be an incomplete stimulation of antigen-specific immune responses, leading to the establishment of persistent infection (Van Reeth et al., 1999).

**FIGURE 2:**
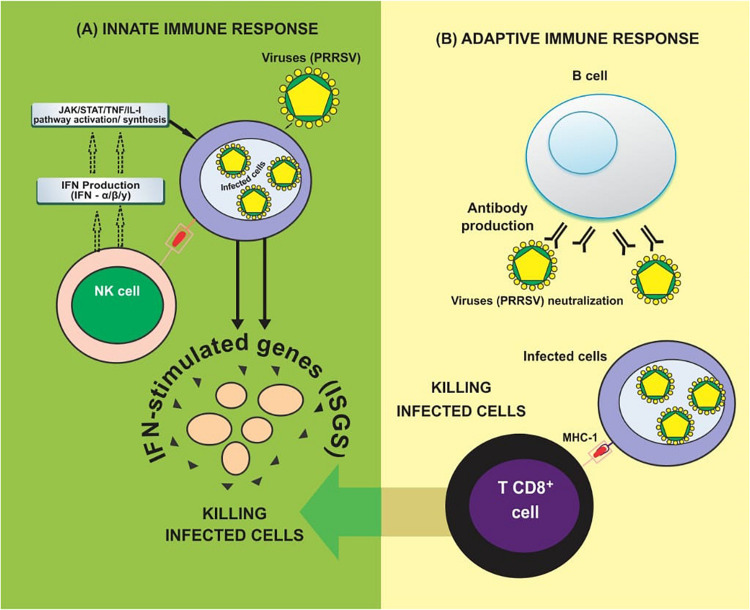
Immune response against viruses. **(A)** Innate immune response: NK cells initially recognize virus infection and evokes antiviral responses by producing Types I & II interferons (IFN-α/β/y). Type I interferons can initiate the activation of JAK/STAT signaling to induce hundreds of IFN-stimulated genes (ISGs), which help to eliminate virus-infected cells. **(B)** Adaptive immune production of antibodies against viral antigens. T CD8^+^ cells secrete cytokines such as TNF-α and IFN-γ which help to eliminate virus-infected cells (Adapted from Carrillo et al., 2017).

Besides, PRRSV is capable of evading type I interferon-mediated antiviral response by several mechanisms including the secretion of IL-10, a potent immunosuppressive cytokine which is capable of downregulating the host immune responses. It is also involved in encoding several IFN antagonists which block either IFN induction or IFN-activated JAK/STAT signaling (Patel et al., 2010; Wang and Zhang, 2014; Yang et al., 2017). Then Chen et al. (2010) stated that the sp1 of PRRSV generates two self-cleaved subunits: nsp1a and nsp1b. They both inhibited IFN-β expression by affecting IRF3-mediated IFN induction (Chen et al., 2010). Nsp2 is the most prominent non-structural protein encoded by PRRSV; it antagonizes IFN induction by blocking IRF3 phosphorylation and nuclear translocation. Researchers have also provided evidence that nsp4 is another IFN antagonist which interferes with the NF-κB signaling pathway through the cleavage of NEMO to weaken the IFN-β induced by poly (I:C) Huang et al. (2014). Furthermore, nsp11 can suppress the activation of IFN-β by cleaving the mRNA of IPS-1 via the endoribonuclease domain (Shi et al., 2011). Accumulating evidence has also reported that other than the nsps of PRRSV, structural proteins, such as N protein, prevent IFN-β induction like that of nsp2 (Sagong and Lee, 2011). Apart from inhibiting IFN induction, PRRSV nsp1b also prevents IFN-activated JAK/STAT signaling via promoting degradation of KPNA1, an essential transporter for mediating the nuclear import of ISGF3 (Patel et al., 2010; Wang et al., 2013a). Moreover, nsp7, nsp12, GP3, and N of PRRSV also interfere with IFN-activated signaling by an unknown mechanism (Wang et al., 2013b; Yang et al., 2017). See Figure 2 for a simplified summary of activities of the innate and adaptive immune system against viral (PRRSV) infection.

Currently, several strategies are being employed in combating PRRSV, including manipulating the antiviral roles of host microRNAs, miRNAs (miRNA-181) small interfering RNAs (siRNAs), short-hairpin RNAs (shRNAs), artificial microRNAs, and PMOs (morpholino oligomers) and vaccines. All these have been tested for their ability to inhibit PRRSV infection both *in vitro* and *in vivo* (Du et al., 2017a). Herein we reviewed the current information on the various antiviral mechanisms of some selected TCMs against PRRSV infection. They were classified into two major groups based on their mode of actions, including those that directly inactivate the virus by targeting a specific step in the PRRSV life cycle (Direct Acting Antivirals), such as PRRSV entry blockers, viral replication inhibitors, assembly, packaging and progeny release inhibitors and those that indirectly inhibit the growth of the virus (Indirect-Acting Antivirals) by promoting the response of the immune system against PRRSV invasion through the regulation of cytokine synthesis (Burns et al., 2010). This group of TCMs act as immunomodulators and inhibitors of metabolic pathways that promote PRRSV pathogenesis.

### Viral Propagation and Replication

Functionally, a virus exists in two unique forms; firstly, as a virion, which is the static, inactive extracellular form, devoid of any metabolic activity, but serves as the carrier of the viral genetic makeup and secondly, as an uncoated dynamic intracellular form, consisting of the full viral genetic material itself. Virus propagation involves the growth and multiplication of viruses through the synthesis of the viral genetic materials and release of the mature virion from infected host cells (*in vivo*) or media (*in vitro*) to new host or media (Faisst, 1999). Virus replication, on the other hand, is the synthesis of complementary negative-sense RNA, which then serves as a template for positive-sense (genome-sense) RNA synthesis (Maclachlan et al., 2016). Thus, to propagate itself, a virus has to enter a living cell and after that, release its genome from its capsid, and interacts with the host cell to replicate and produce viral proteins. New capsids are assembled, the newly synthesized genomes are then packaged into these capsids either concomitantly with or after their assembly. This results in progeny virions, which are released from the cell and transferred to new host cells. They achieve this by hijacking the biochemistry and metabolic pathways of the host cell to produce multiple copies of themselves and thus continue to infect new hosts.

### Life Cycle of PRRSV

Viral entry and uncoating trigger the commencement of the life cycle, which encompasses genome translation and replication, leading to the assembly and release of infectious viral progeny. The entire life cycle begins with the translation of the large replicase gene. This replicase gene consists of two large open reading frames (ORFs) that are connected by a ribosomal frameshift site leading to an ORF1a encoded polypeptide (pp1a), and an ORF1a and 1b encoded polypeptide (pp1ab). This phase is followed by the translation of both pp1a and pp1ab polyproteins and then extensive autoproteolytic processing which leads to the synthesis of at least about 13 non-structural proteins (Pasternak et al., 2006). Upon proteolytic processing, the non-structural proteins become associated with intracellular membranes to form the replication/transcription complex (RTC) that is responsible for the synthesis of viral RNA. It comprises of the synthesis of the nested set of mRNAs and replication of the viral genomic RNA. RTC makes use of endoplasmic reticulum (ER) membranes to induce the formation of double-membrane vesicles (DMV) which establishes the RTC (Faaberg, 2008). A full-length negative-strand RNA (also called antigenome) is then synthesized by the RNA-dependent RNA polymerase (RdRp) which it uses as the template for the synthesis of new genomic RNA. In addition to its replication, the PRRSV genome also functions as a template for synthesizing sub genomic (sg) mRNAs generated from negative-strand intermediates. The transcripts of sg help to express the seven structural proteins of PRRSV. The translation of the structural proteins which takes place on ribosomes of the ER is followed by the phosphorylation of the N protein and the formation of homodimers that enclose the newly synthesized full-length genomes (Wootton et al., 2002; Wootton and Yoo, 2003). In the ER, the major glycoprotein; GP5 and the minor glycoproteins GP2, GP3 and GP4 are glycosylated.

**FIGURE 3:**
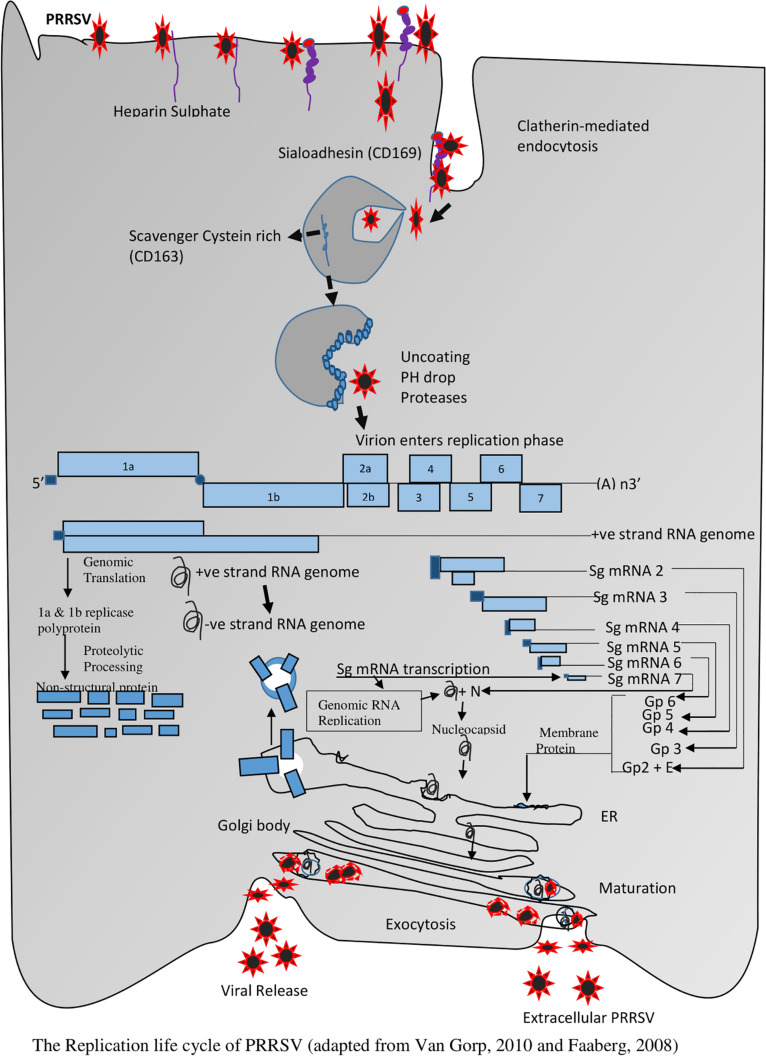
The Replication life cycle of PRRSV (adapted from Faaberg, 2008 and Van Gorp et al., 2010).

At the same time, the major envelope proteins form an M-GP5 complex heterodimer via an intermolecular disulfide bridge by engaging the cysteine residues in the ectodomains of both proteins (Mardassi et al., 1996), while the minor envelope proteins GP2, GP3, and GP4 form a heterotrimer complex in the PRRSV envelope (Wissink et al., 2005) in a non-covalent union of GP2:GP4: GP3 in a 1:1:1 ratio. When all structural components are located at the same site of the ER, the nucleocapsid moves around the membrane, and budding eventually takes place in the lumen of the ER. The newly formed miniature virions move through the Golgi apparatus, and the process of maturation takes place via trimming of the core high-mannose glycan residues on the glycoproteins, and they are replaced with complex sugars. The GP2-GP4 heterodimer may become covalently attached to GP3 via disulfide linkages in the Golgi apparatus, as in the case of EAV. The newly synthesized PRRSV virions are finally released into the extracellular space via exocytosis (Baker and Denison, 2008; Faaberg, 2008; Van Hemert and Snijder, 2008), see Figure 3.

## Traditional Chinese Medicines (TCM)

Traditional Chinese Medicines have proven to be a valuable source of therapeutic herbs in China and other parts of the world. They elicit multiple biological activities such as antiviral, antibacterial, anti-inflammatory and antioxidant (Chen and Yu, 1999), for example, the TCM “Shuanghuanglian” has been used as an antiviral and antibiotic drug to treat respiratory-related diseases in China since 1973 (Zhang et al., 2013), its chemical components include chlorogenic acid, baicalin, and forsythia glycosides (Zhang et al., 2013); “Artemisinin” has been famous for its anti-malaria activity since 1979 (Tu, 2011). The discoverer Youyou Tu was awarded the Nobel Prize for Physiology or Medicine in 2015 (Su and Miller, 2015). This gave more credence to the efficacy of TCM as a significant source of potential alternative therapy against diseases. Investigations from previous and on-going researches based on advanced molecular and analytical techniques have led to the extraction, quantification and classification of most of the bioactive compounds in TCM, including several classes of glycosides, terpenoids, coumarins, isoflavones, alkaloids, flavones, phenolic acids, lignans, lignins, tannins, carotenoids, polyacetylenes, asiatic acid, madecassoside, madecassic acid, asiaticoside, polyacetylene, among others. These compounds have various antiviral and immune-modulatory effects (Duan et al., 2015; Ge et al., 2018).

Some TCMs have been reported to have potent antiviral activities against PRRSV based on different antiviral strategies (Wang et al., 2003; Ma et al., 2013; Yang et al., 2013; Sun et al., 2014a; Ge et al., 2018), including those with direct virucidal/antiviral mechanisms (Direct acting antivirals) and those that inhibit the proliferation of the virus by an indirect mode of action through the tweaking of the host innate immune system (Indirect acting antivirals).

## Direct-Acting Antivirals (DAAs)

Direct-acting antivirals (DAAs) are drugs that target specific steps in the life cycle of a virus and thus alters the viral genome and proteins (Aqeel, 2017; Holmes et al., 2019). Although, just like the Indirect acting antivirals, DAA is a term for drugs designed for the treatment of HCV infection (Vachon and Dieterich, 2011; Blach et al., 2017; Amer, 2018; Darrick and Chung, 2019), however, in this review, TCMs with antiviral strategies that target specific steps in the life cycle of PRRSV have been classified under this group. They include PRRSV entry blockers, replication inhibitors, and PRRSV packaging, assembly and viral particles release inhibitors.

### PRRSV Entry Blockers

As an enveloped virus, PRRSV enters into PAMs through a pH-dependent (low pH) membrane-bound, receptor-mediated endocytosis (Kielian and Jungerwirth, 1990; Kreutz and Ackermann, 1996; Kim et al., 2006; Ryu, 2017; Samer and Shyamasundaran, 2020). Several bioactive compounds extracted from Chinese herbs exhibit antagonistic activities toward PRRSV, or prevent the viruses from attaching to putative receptors, or block their entrance into cells *in vitro* by interfering with the process that leads to the uncoating of the viral genome (Figure 3). These happen either by (i) bioactive compound - viral interaction, (ii) increasing the pH level of the endocytic pathways, or (iii) by modulating the expression of putative receptors. Some of these mechanisms are outlined below.

### Interaction With the Receptor to Interfere With Viral Attachment

Viral attachment and entry into host cells is the first stage of the viral life cycle. PRRSV infection is dependent on various cellular receptors or factors (Du et al., 2017a). Numerous studies have demonstrated that PRRSV entry blockers are a promising novel approach to combat PRRSV infection, these blockers include antibodies from vaccines, some chemical reagents and some TCMs (Du et al., 2017a). (–)-Epigallocatechin-3-gallate (EGCG) is the most abundant bioactive compound in Green tea (Friedman, 2007; Chacko et al., 2010; Steinmann et al., 2013), it has been reported to inhibit PRRSV replication in MARC-145 cells *in vitro* based on the following multiple mechanisms: (i) it prevented the attachment of PRRSV to susceptible cells by downregulating the expression of PRRSV receptors CD163, MYH9, and HS in EGCG pre-treated cells in a dose-time dependent manner; (ii) it acted directly on the visions as an antiviral receptor mimetic which competes with both heparin and sialic acid moieties for virion binding by attaching to the active site of the PRRSV glycoprotein (GP5) (Colpitts and Schang, 2014; Ge et al., 2018).

### Interaction With Cellular Membrane to Inhibit Cellular Penetration by PRRSV Particles

Viral proliferation can be inhibited by interfering with the cellular membranes of host cells. Several studies have revealed that some TCM can interact with the cellular membranes of macrophages thereby inhibiting cellular penetration by viral particles, notable among these viruses include HBV (Sato et al., 1996), HCV (Matsumoto et al., 2013), HSV (Huang et al., 2012), SARS coronavirus (Cinatl et al., 2003), and influenza viruses (Wolkerstorfer et al., 2009). Glycyrrhizin (GL), a triterpene saponin found in abundant quantity in licorice root (*Glycyrrhiza glabra*), which has been reported to be an active antiviral bioactive compound against the various stages of the life cycle of PRRSV *in vitro*. The postulated antiviral mechanisms of GL include direct effects on the adsorption, penetration and particle maturation steps of PRRSV reproductive cycle. It was reported that PRRSV-infected MARC-145 cells treated with GL during the penetration phase of the viral life cycle showed a significant reduction in PRRSV particles observed at this stage of the viral life cycle. This demonstrated that GL inhibited PRRSV internalization into MARC-145 cells (Duan et al., 2015). Besides, earlier studies had revealed that GL inhibits virus penetration by modifying the viscosity of the phospholipid content of the lipid bilayer of viral cell membranes. PRRSV enters into MARC-145 cells through a microfilament-dependent endocytic pathway (Kreutz and Ackermann, 1996). It was, therefore, inferred that GL might most likely inhibit the penetration of PRRSV by interfering with the cellular membrane and eventually lead to a reduction in endocytosis (Duan et al., 2015). However, further studies will be needed to verify this claim.

### Inhibition of Virus Internalization and Cell Fusion

An enveloped virus fuses its membrane with a host cell membrane, thereby releasing its genome into the cytoplasm and initiating the viral replication cycle. Some TCM stops the replication cycle of viruses by preventing virus internalization and virus-mediated cell fusion. After internalization, the viral particles are transported to the endosome. The acidic condition in the endocytic pathway causes changes in the conformation of the viral membrane and glycoproteins, which result in the uncoating of virus particles, thereby causing infection.

For PRRSV, the process can be inhibited by curcumin due to the inhibition of PRRSV membrane fusion, N protein and viral progeny production in MARC-145 and PAM cells (Du et al., 2017b). Curcumin, also known as diferuloylmethane, is a natural bioactive compound isolated from the rhizomes of *Curcuma longa* (Du et al., 2017b). Curcumin belongs to the polyphenol group of organic compounds and has multiple functions including its antiviral ability (Venkatesan, 1998; Venkatesan et al., 2000; Arun and Nalini, 2002; Mahady et al., 2002; Aggarwal et al., 2003; Kim et al., 2003; Reddy et al., 2005; Moghadamtousi et al., 2014; Mathew and Hsu, 2018; Praditya et al., 2019). In a study involving the treatment of infected MARC-145 and PAM with Curcumin, curcumin significantly inhibited the entry of PRRSV into the cells by interfering with all the post-internalization stages of PRRSV, which blocked viral membrane fusion and stopped the uncoating of the PRRSV genome in MARC-145 and PAM cells (Du et al., 2017b).

Tetrahydroaltersolanol C (TD-C) was also reported to inhibit the internalization stage of PRRSV. TD-C is a hydroanthraquinone extracted from *Alternaria* sp., a fungus obtained from *Sarcophyton* sp. (a soft coral found in tropical and subtropical seas) (Garozzo et al., 2011; Zhang et al., 2016). TD-C significantly inhibited the proliferation of PRRSV when cells were pre-incubated or co-incubated with it; the significant inhibition of the yield of viruses proved that it might effectively interfere with viral internalization and cause a significant reduction in the viral progeny titers. TD-C significantly exhibited antiviral activity against PRRSV during viral internalization and replication stages. The mechanism of action could be attributed to the direct inhibition of growth, adsorption, fusion, internalization, and replication of PRRSV, which are all critical stages of the life cycle of the virus (Kuo et al., 2010; Garozzo et al., 2011). However, the specific mode of action of TD-C against PRRSV still needs further investigation.

### Prevention of PRRSV Internalization Through Viral Particles Agglutination

The entry phase of PRRSV is made up of early viral attachment and internalization (Gao et al., 2013). Just like other enveloped viruses, PRRSV infects cells after attachment to a cellular receptor, and it is internalized from the surface of susceptible cells within 3–6 h (Gao et al., 2013). *Cryptoporus volvatus* (Pk.) Hubbard is a polypore fungus that belongs to the family Polyporaceae. Aqueous extracts of *C. volvatus* inhibited PRRSV internalization but not its attachment to the host cells by causing the viral particles to agglutinate and form clusters outside the cell membrane (Gao et al., 2013). Other secondary effects, including the inhibition of PRRSV protein synthesis, infectious PRRSV particle release and the cell-to-cell spread of the virus were reported due to the inhibition of PRRSV entry and RNA polymerase activity. The aqueous extract obtained from *C. volvatus* consist of numbers of bioactive components and the exalt active ingredient responsible for its antiviral activity is under investigation.

Besides, the presence of PRRSV in host cells can be inhibited through multiple mechanisms, for example, aromatic plants, such as *Thymus vulgaris* which is rich in essential oils and lipophilic substances (Nikolic et al., 2014), and *Nepeta cataria, whose* bioactive compounds include monoterpenes, sesquiterpenes, diterpenes, triterpenes, flavonoids, phenol, essential oils, and others (Adiguzel et al., 2009); can both significantly reduce PRRSV amount at the pre-entry and post-entry steps of the viral life cycle by blocking viral attachment, adsorption, replication, and release (Kaewprom et al., 2017). A summary of the PRRSV entry blockers described above is presented in Table 1.

**TABLE 1:** A summary of PRRSV entry blockers.

TCM	Compounds	Antiviral Strategy	Mechanism	References
Green Tea (*Camellia sinensis*)	-(-) epigallocatechin-3-gallate (EGCG)	Inhibits viral attachment and penetration	Down-regulate the expression of PRRSV receptors Acts as a receptor mimetic by binding to viral glycoprotein and interfere with viral attachment	Ge et al., 2018
Licorice roots (*Glycyrrhiza glabra*).	Glycyrrhizin (GL)	Inhibits viral penetration and internalization	Modifies the viscosity of the phospholipid content of the lipid bilayer of viral cell membranes	Duan et al., 2015
*Thymus vulgaris*	*Thymus vulgaris* hydrosol	Blocks pre-entry and post-entry stages of PRRSV	Binds to PRRSV receptors, interferes with viral envelope and masks viral components	Kaewprom et al., 2017
*Nepeta cataria*	*Nepeta cataria* hydrosol	Blocks pre-entry and post-entry stages of PRRSV	Blocks viral attachment and adsorption	Kaewprom et al., 2017
Turmeric rhizomes *(Curcuma longa)*	Curcumin	Blocks viral membrane fusion and stops the uncoating of PRRSV genome	Prevents virus internalization and virus-mediated cell fusion by preventing the reduction in cellular pH level	Du et al., 2017b
Tea Seed	Tea Seed Saponins (TS)	Blocks PRRSV entry and inhibits viral replication	Direct inactivation of virus	Li E. et al., 2015
*Cryptoporus volvatus* (Pk.) Hubbard	*Cryptoporus volvatus* extract	Inhibit PRRSV internalization but not attachment to the host cells	Inhibits PRRSV RNA-dependent RNA polymerase (*RdRp*)	Gao et al., 2013
*Alternaria* sp. (Fungus)	Tetrahydroaltersolanol C (TD-C)	Interferes with viral internalization	Direct inhibition of growth, adsorption, fusion, internalization and replication	Zhang et al., 2016

### PRRSV Replication Inhibitors

The replication stage is the most significant phase in the life cycle of a virus. It starts by the translation of the large replicase gene and through a series of synthetic pathways culminate in the viral particles’ synthesis and release. Inhibitors of viral proteases, polymerases, integrases (helicases), reverse transcriptase and heparanase of several viruses have been successfully investigated, including the inhibition of different viral stages in *in vitro* and clinical trials (Li and Peng, 2013).

### Inhibition of the Early Stage of PRRSV Replication Through Viral Inactivation

Host cells are usually less susceptible to inactivated viruses, and the process includes solvent/detergent, low pH, and heat treatments (Sandle, 2015). Chlorogenic acid is a vital plant polyphenol derived from coffee. It is an ester of caffeic acid (Trugo, 2003; Cheng et al., 2013). Chlorogenic acid has a strong anti-PRRSV effect *in vitro* (Wang et al., 2008).

Similar results were observed in Scutellarin-treated cells infected with PRRSV. Scutellarin is a flavone glucuronide derived from *Scutellaria barbata* and *Scutellaria lateriflora*. Its pharmacological actions include anti-inflammation and viral inhibition. They inhibited the replication of PRRSV by directly inactivating PRRSV *in vitro* Cheng et al., 2013). A similar effect was also observed in infected Marc-145 cells treated with chlorogenic acid and scutellarin at different time intervals (Cheng et al., 2013).

*Cynodon dactylon* is a common perennial native grass that grows naturally in the tropics and warm temperate regions. It is also known as Bermuda grass and belongs to the family Poaceae. The extracts of *C. dactylon* are known for their antimicrobial, anti-diabetic, anti-hyperlipidemia, anti-inflammatory, antiemetic and cardioprotective activities (Singh et al., 2007; Garjani et al., 2009; Immanuel et al., 2009). A virustatic and virucidal study was conducted to investigate its effect on the replication stage of PRRSV; the results demonstrated that its ethanol extracts had both virustatic and virucidal effects on the replication stage of PRRSV. It significantly inhibited the replication of the virus in a dose-dependent manner. IPMA also revealed that the same concentration of ethanol extracts of *C. dactylon* significantly inactivated the already released virions (Pringproa et al., 2014).

### Inhibition of N Gene Expression and N-Proteins Synthesis

As the most abundant viral structural protein, PRRSV nucleocapsid (N) protein form the viral capsid by interacting with itself through covalent and non-covalent interactions to package the viral genome (Liu et al., 2016). Inhibition of N gene expression or N-proteins synthesis interferes with PRRSV replication. This occurs by down-regulating the N gene expression both at the mRNA stage and protein level. The expression of N-Protein leads to the expression of TNF-α, a proinflammatory cytokine and the activation of NF-κB pathway with a concomitant up-regulation of Interleukin-10 (IL-10) in PAMs (Wongyanin et al., 2012; Song et al., 2013). IL-10 is pleiotropic, and it is produced by many types of cells, including monocytes/macrophages, cells that play a critical role in the inflammatory process (Moore et al., 2001). The up-regulation of IL-10 inhibits expression of N-Protein, prevents TNF-α-induced apoptosis by downregulating IKK phosphorylation and in turn, NF-κB activation (Dhingra et al., 2009). Sodium tanshinone IIA sulfonate (STS) is a bioactive compound derived from *Salvia miltiorrhiza* (Sun et al., 2012). The anti-PRRSV activity mechanism of STS involves two pathways: firstly, it inhibits the transcription and translocation of N-protein, and thereby reduce the N protein-induced host cell apoptosis (Sun et al., 2014a); secondly, STS inactivates PRRSV directly.

Findings from RT-PCR, western blot and indirect immunofluorescence assays revealed that the biological activity of STS is dose-dependent.

Flavaspidic acid AB (FA-AB) was also observed to inhibit the replication stage of PRRSV by inhibiting the synthesis of N-proteins in FA-AB treated PAM cells (Yang et al., 2013). FA-AB belongs to the family of phloroglucinol according to its chemical structure, which is isolated from *Dryopteris crassirhizoma* Nakai (a semi-evergreen fern) and possesses anti-oxidant, anti-bacterial and anti-tumor (Kapadia et al., 1996; Mathekga et al., 2000; Lee et al., 2003; Gupta et al., 2010; Vo and Kim, 2010). Yang et al. (2013) reported that FA-AB partially inhibited the entry stage of PRRSV, blocks the cell-to-cell transmission pathway which is typical of PRRSV and caused a reduction of virus titers in cells by inhibiting the synthesis of N-proteins in a dose and time-dependent manner.

### Zn^2+^ From Natural Zinc Ionophore Inhibits PRRSV Replication by Decreasing the Transcription and Translation Levels of PRRSV N Protein

Pyrithione is a common organosulfur compound with molecular formula C_5_H_5_NOS. Pyrithione is the short form of pyridinethione. This compound is a natural zinc ionophore, and it functions as a centrosymmetric dimmer to increase intracellular Zn^2+^ concentration via its oxygen and sulfur centers (Qiu et al., 2013). It is found in Persian shallot-an Asian species of onion native to central and southwestern Asia (Ebrahimi et al., 2009). It exists naturally as a pair of tautomers. The major and minor forms are thione 1-hydroxy-2(1H)-pyridinethione and thiol 2-mercaptopyridine N-oxide, respectively. Zinc ions are involved in many critical cellular processes. They play a significant role in the proper folding and activity of various cellular enzymes and transcription factors. Keeping the available Zn^2+^ concentration in cells at a stable homeostatic equilibrium is very vital for cell survival because any change above or below the normal threshold levels can trigger apoptosis in a variety of cell types (Velthuis et al., 2010). It is also an essential cofactor for numerous viral proteins (Qiu et al., 2013). A mechanistic study has shown that PT has potent antiviral activity against the replication stage of the life cycle of PRRSV and other several RNA viruses (Guo et al., 2017). It does this by effectively decreasing the transcription and translation levels of the PRRSV N protein with the help of the imported extracellular Zn^2+^.

### Inhibition of the Post Entry Stage of PRRSV Replication by Inhibiting the N-Protein Synthesis and P13K/Akt and ERK Signaling Pathways

Porcine reproductive and respiratory syndrome virus RNA replication begins at the post-entry stage of viral replication, an antiviral strategy based on inhibition of the post-entry stage of PRRSV replication has also been reported (Zhang et al., 2016). Isobavachalcone (IBC) is one of the prenylated chalcones derived from *Psoralea corylifolia* (Kuete and Sandjo, 2012); it belongs to the subclass of flavonoids and has a broad-spectrum biological activity against bacteria and fungi, and as well acts as an anti-reverse transcriptase, anti-tubercular and antioxidant (Kuete and Sandjo, 2012). Marc145 cells were infected with PRRSV and then treated with IBC at specific time points (2, 6, 12, or 24 h), the levels of PRRSV replication were inhibited from 2 to 6 hpi, demonstrating that IBC blocks the early stages of the viral life cycle, most likely at the initiation of viral RNA replication. Further verification using IBC treated PAMs cells showed that the dsRNA levels were significantly reduced (Wang et al., 2018).

Extracts from *Sasa quelpaertensis* Nakai, (*Jeju-Joritdae*) leaves (SQE), a dwarf type of bamboo grass that is widely distributed on Mt. Halla in Jeju Island, South Korea have also been reported to show potent biological activities against PRRSV. It was observed that SQE significantly inhibited the post-entry stages during the replication of PRRSV in treated cells. This was demonstrated by reduced progeny production and inhibition of viral protein expression. The anti-PRRSV strategy of SQE is based on its ability to inhibit the synthesis of genomic RNA and sg mRNA (Kang and Lee, 2015).

### Regulation of Viral ORF1 Function

Porcine reproductive and respiratory syndrome virus ORF1 encodes 14 non-structural proteins, which include four proteases (NSP1, NSP1, NSP2, and NSP4), the RNA-dependent RNA polymerase (NSP9), a helicase (NSP10) and an endonuclease (NSP11) (Boon et al., 1995; Snijder and Meulenberg, 1998; Ziebuhr et al., 2000; van Aken et al., 2006). Dipotassium glycerrhetate (DG) is a derivative of glycyrrehetinic acid (GA) extracted from the roots of Licorice (*Glycyrrhiza glabra*), it inhibits the replication of PRRSV by regulating the function of the viral ORF1 and concomitantly inhibiting the synthesis of RNA-dependent RNA polymerase (RdRp) (Wang et al., 2013). DG protected cells against PRRSV induced apoptosis through the inhibition of Caspase-3 protein degradation system after been used to treat infected cell (Wang et al., 2013).

### Inhibition of Viral Nsp9-p107 Interaction

The Nsp9 of PRRSV contains the viral replicase RNA-dependent RNA polymerases (RdRp), it plays a critical role in the replication of the virus (Lehmann et al., 2016; Sun et al., 2018). The SDD (Serine-Aspartic Acid-Aspartic acid) motif, located at residues 3050–3052 of Nsp9, has been reported to be vital for its polymerase activity and virus replication (Zhou et al., 2011). Some researchers have reported that a host of proteins interact with Nsp9 and influence the replication of the virus (Dong et al., 2014; Li et al., 2014; Zhao et al., 2015). PRRSV could be inhibited by specific siRNA targeting Nsp9 gene (Xie et al., 2014). Sun et al. (2018) used the dual-luciferase reporter assay to detect the interaction between Nsp9 and the E2F reaction promoter p107, and the results indicated that Nsp9 could activate the promoter p107, and consequently aid cells to enter the S phase, this enhances the replication of the virus. Chinese traditional herbal medicine, matrine, an alkaloid that is extracted from *Sophora flavescens Ait*, is an essential herb with a lot of biological activities (Sun et al., 2014b; Yong et al., 2015; Wu et al., 2016). It inactivates PRRSV by direct interaction with the virus, leading to the inhibition of the replication stage of the viral life cycle (Sun et al., 2014b). The effects of matrine on the mRNA expression of PRRSV N gene and the protein expression of pERK1/2 and PRRSV Nsp9 activity was investigated; it was observed that matrine inhibited the interaction between Nsp9 and promoter p107. PRRSV Nsp9 binds to promoter p107 for PRRSV RNA synthesis. Thus, the inhibition of the Nsp9-p107 interaction leads to the termination of the synthesis of PRRSV RNA and related proteins in host cells (Sun et al., 2018), suggesting that the mechanism by which matrine inhibited the replication of PRRSV is independent of the entry process of PRRSV, as well as ERK1/2 signal pathway.

### Inhibition of PRRSV Replication by Interfering With RNA-Dependent RNA Polymerase (RdRp) Activity

The formation of RNA-template or RNA synthesis catalyzed by viral RNA-dependent RNA polymerase (RdRp) is required to replicate and transcribe the viral genome (Kappes and Faaberg, 2015). Inhibitors targeting the viral RdRp were developed as another antiviral strategy to inhibit the replication of positive-strand RNA viruses (Tomei et al., 2003; Gastaminza et al., 2010), for example, several structural classes of non-nucleoside inhibitors (NNIs) can inhibit the non-structural protein 5B RNA-dependent RNA polymerase (NS5B RdRp) of HCV by binding to the protein surface in the narrow cleft in the thumb domain blocking the RdRp activity before the formation of an elongation complex and therefore efficiently block the replication of HCV subgenomic replicons in tissue culture (Tomei et al., 2003; Wang et al., 2003; Lindenbach et al., 2005; Pietschmann et al., 2006; Ban et al., 2013). Nanomolar inhibitors consisting of a class of acyl sulfonamide-thiophene compounds with nanomolar inhibitory effects against the *de novo* initiation activities of DENV1-4 RdRp has also been developed (Lim et al., 2018). This same strategy is currently being explored in combating the current global outbreak of severe acute respiratory syndrome coronavirus 2 (SARS-CoV-2) (Gordon et al., 2020a, b; Yin et al., 2020). Investigation by Gao et al. (2013) revealed that aqueous extracts from *C. volvatus* the inhibited the replication of PRRSV by targeting and interfering with the activity of viral RdRp. *In vivo* study also revealed that aqueous extracts from *Cryptoporus volvatus* (Pk.) Hubbard inhibited the replication of the highly pathogenic PRRSV (HP-PRRSV) in pigs (Gao et al., 2013). Table 2 summarizes this group of compounds and their antiviral strategies.

**TABLE 2:** A summary of PRRSV replication blockers.

TCMs	Compounds	Antiviral strategy	Mechanism	References
*Scutellaria barbata*, *S. lateriflora*	Scutellarin	Inactivates and/or interferes with the early stage of PRRSV replication	Direct inactivation of PRRSV (Virucidal effect)	Cheng et al., 2013
Rhizomes of *Dryopteris crassirhizoma* Nakai	Flavaspidic Acid Ab (Fa-Ab)	Partially inhibits viral entry and significantly Blocks viral replication	Blocks cell-cell spreading Inhibits the synthesis of N-proteins	Yang et al., 2013
Coffee	Chlorogenic acid	Inhibits early stage of the PRRSV replication cycle	Direct inactivation of PRRSV (Virucidal effect)	Cheng et al., 2013
*Cynodon dactylon*	*Cynodon dactylon* extracts	Inhibits PRRSV replication cycle	Virustatic and virucidal effects on the replication stage of PRRSV	Pringproa et al., 2014
*Psoralea corylifolia*	Isobavachalcone (IBC)	Inhibits the Post entry stage of PRRSV replication	Reduces PRRSV N-protein	Wang et al., 2018
*Salvia miltiorrhiza*	Sodium tanshinone IIA Sulfonate	Inhibits PRRSV replication	Inactivates or disrupts the replication pathway of the PRRSV Down-regulates N gene expression and inhibits the transcription, and translocation of N-protein.	Sun et al., 2014a
Licorice roots (*Glycyrrhiza glabra*).	Dipotassium Glycerrhetate (DG)	Inhibits the replication of PRRSV	It suppresses the function of ORF1	Wang et al., 2013
*Persian shallot*	Pyrithione	Inhibits PRRSV replication	Decreases the transcription and translation levels of the PRRSV N protein with the help of imported extracellular Zn^2+^	Guo et al., 2017
*Sasa quelpaertensis* Nakai	*Sasa quelpaertensis* (SQE)	Inhibits the post-entry steps during the replication of PRRSV	Progeny reduction, diminished viral protein expression, and reduced synthesis of genomic RNA, and sg mRNA	Kang and Lee, 2015
*Sophora flavescens Ait*	Matrine	Inhibits the replication of PRRSV	Inhibits the interaction between Nsp9, and promoter p107	Sun et al., 2019

### PRRSV Packaging, Assembly and Release Inhibitors

Porcine reproductive and respiratory syndrome virus package and assembly stage starts after 8 h-post infection (hpi). This is the last stage of the viral life cycle. During this stage, the newly synthesized viral genomes are packaged into capsids for intracellular transport, enveloped and then finally released into the extracellular space via exocytosis (Pol et al., 1997; Snijder, 1998; Nauwynck et al., 1999; Duan et al., 2015; Kappes and Faaberg, 2015; Guo et al., 2017). Available evidence has proved that bioactive compounds from some TCMs interfere with the packaging, assembly, and release of PRRSV particles. More investigations are needed to confirm this claim further.

### Inhibition of PRRSV Progeny Release by Regulating the Expression of Cytokines

The initial primary defense response of animals to viral infection is the production of Interferon-alpha (IFN-α) (a type 1 interferon), often followed by inflammatory cytokines such as tumor necrosis factor (TNF) and interleukin-1 (IL-1). However, with PRRSV infection, such response is weakened or significantly suppressed. Several reporters have stated that the expression of interferon –α inhibits the replication of PRRSV *in vitro* (Brockmeier et al., 2012, 2017). The expression of inflammatory cytokine plays a very significant role in the organization of the host immune system against a variety of viral infection (Balasch et al., 2019). TNF, IL-1β, and IL-6 are important activators of the nuclear transcription factor, NF-κB (DiDonato et al., 1997; Christman et al., 2000). The function of NF-κB during infection is to regulate the transcription of hundreds of genes, including antigen receptors, inflammatory and immunoregulatory cytokines, adhesion molecules, inhibitors of apoptosis, acute phase proteins, and innate effector molecules. It was speculated that the mild or subclinical respiratory abnormalities involved in PRRSV infection might be due to the lack of activation of NF-κB. However, when there is a lack of an acute inflammatory response and weak innate antiviral activity, the resultant effect might also be an incomplete stimulation of antigen-specific immune responses, and this will promote the establishment of persistent infection (Van Reeth et al., 1999). *Platycodon grandiflorum* A. DC (Campanulaceae) is a well-known Chinese herb. The root of this herb is a significant source of saponins (Xie et al., 2008; Zhang et al., 2018a), it is used as an expectorant for pulmonary diseases and a therapy for respiratory disorders (Lei et al., 2018). Among the Saponins present in *Platycodon grandiflorum* A. DC, is Platycodin D (PD), an oleanane-type triterpenoid saponin with two sugar chains attached to positions C-3 and C-28 of aglycone. It is regarded as the most biologically potent among *platycodin saponins* (Xie et al., 2009). PD impaired the entry and replication of PRRSV in MARC-145 cells in a dose-dependent manner. A significant decrease in the number of viral progenies was observed both in MARC-145 cells and PAMs (Zhang et al., 2018a) in treated infected cells.

Platycodin D treatment significantly inhibited the expression of six cytokines, including IFN-α IFN-β, IL-1α, IL-6, IL-8, and TNF-α in a time-dependent manner in infected cells (Zhang et al., 2018a). Interestingly, it is worthy to note that PD also enhanced TNF-α expression in un-infected PAMs indicating that PD has a dual antiviral activity by increasing the expression of TNF-α in uninfected cells thereby enhancing or fortifying the innate immune system of cells against invading PRRSV from neighboring cells, on the one hand, and concomitantly, reduces the high expression of TNF-α in infected cells, on the other hand, thereby helping the infected cells to attenuate TNF-α induced apoptosis. This pleiotropic property of PD might be indirectly responsible for the inhibition of PRRSV progeny release in PD treated cells (Zhang et al., 2018a).

### Deactivation of NF-κB/Heparanase Pathway

Heparan sulfate is an integral part of the extracellular matrix (ECM); it enhances the initial attachment of viruses to target cells. Heparanase is the only enzyme that can degrade HS and thus, promote the release of HS-bound viruses, cytokines and other related proteins such as growth factors in many biochemical paradigms (Yang Y. et al., 2015). As a receptor of PRRSV, HS bind to PRRSV, thus a decrease in the availability of HS at surfaces of cells encourage PRRSV exit. Studies have revealed that heparanase is upregulated through the activation of NF-κB pathway (by TNF, IL-1β, IL-6, and other vital activators) when cells are infected by viruses, which is then translocated to the cell surface, leading to the decreased HS expression. This permits the release of exiting viral particles (Hadigal et al., 2015).

Pyrithione (PT) is a broad-spectrum antimicrobial compound. By the same biochemical mechanism with which it inhibited the replication of PRRSV, it also concomitantly stopped the packaging, assembly, and release of the viral particles via multiple pathways. By interfering with NF-κB activation, PT inhibited the expression of heparanase and then blocked the loss of HS from the cell surface, thereby, stopping the release of virus and cytokines (Guo et al., 2017). The expression of heparanase is increased in PRRSV infected cells; this resulted in a remarkable decrease in the level of HS during the later stage of PRRSV infection. And interestingly, treatment with PT caused a significant increase in the level of HS present on the surface of PRRSV infected cells. The increased level of HS led to a significant decrease in the number of viral progeny released (Guo et al., 2017). See the summary of PRRSV packaging and assembly inhibitors in Table 3.

**TABLE 3:** A summary of PRRSV packaging and assembly inhibitors.

TCMs	Compounds	Antiviral Strategy	Mechanism	References
*Platycodon grandiflorum* A. DC	Platycodin D (PD)	Multiple strategies: Blocks viral entry and internalization, inhibits viral replication, and stopped viral release	Binds to one of the PRRSV surface molecules (E and GP2 to GP5) Inhibits PRRSV NSP9 RNA levels, and N protein in a dose dependent manner Enhances TNF-α expression	Zhang et al., 2018a
Grape seed *Vitis vinifera*	Proanthocyanidin A2 (PA2)	Multiple Strategies: Blocks viral attachment, internalization, viral packaging, and progeny release	Direct PRRSV inactivation or impairment. Inhibits PRRSV NSP9 RNA expression in a dose dependent manner Inhibits the expression of PRRSV – induced pro-inflammatory cytokines Inhibits the release of PRRSV progeny particles by deactivating the NF-κB pathway	Zhang et al., 2018b
*Persian shallot*	Pyrithione	Stopped the packaging, assembly, and release of the viral particles via multiple pathways	Interferes with NF-κB activation Inhibits the expression of heparanase, and then blocked the loss of HS from the cell surface	Guo et al., 2017

## Indirect Acting Antivirals (IAAs)

These group of antivirals inhibit the growth of viruses through the modification of the host immune system. They inactivate viral genomes and proteins through the regulation of cytokine synthesis, lipid metabolism and immune cells activation (Burns et al., 2010) and thus, abrogate the pathways hijacked by viruses to perpetuate infection (Aqeel, 2017) without compromising the host cell metabolism. Categorized under this group are TCMs that inactivate PRRSV genome and proteins by modulating the host immune system and deactivating metabolic pathways that promote PRRSV pathogenicity (Zhang et al., 2018b).

### Immunomodulators and PRRSV Pathogenic Pathways Inhibitors

The first line of the host defense against viral infections is the innate immune system; it consists of a network of barriers, including physical barriers, i.e., the skin and mucous membranes; chemical barriers, i.e., antimicrobial peptides, pH, lipids, enzymes, as well as immune cells, i.e., monocytes, macrophages, eosinophils, neutrophils, and natural killer (NK) cells (Lunney et al., 2016). The structural and functional integrity of the host immune system guarantees the initiation of an immune response against intracellular pathogens, prevent the replication of viruses in host cells and their invasion into mucosal tissues (Koyama et al., 2008). PRRSV infection affects both innate and adaptive immune responses, i.e., delays and reduces the humoral and cell-mediated immune (CMI) responses, deregulates cytokines expression (Renukaradhya et al., 2010; Dwivedi et al., 2011; Liu et al., 2011), and induces prolonged viremia and persistent infection in pigs (Molina et al., 2008). The immune-related functions of TCMs with anti-PRRSV effect are associated with their ability to regulate the production of cytokines in host cells (Burns et al., 2010) and deactivate pathways that promote PRSSV pathogenesis (Zhang et al., 2018b).

These soluble extracellular proteins or glycoproteins are essential regulators of biochemical processes that occur in the intercellular domains; they help to mobilize cells engaged in innate and adaptive inflammatory host defense mechanisms such as the regulation of NK cell function during viral infection which is regulated by IFNα, IFNβ, IL-12, and IL-15 (Nguyen et al., 2002), cell growth, differentiation, cell death, angiogenesis, as well as developmental and repair processes aimed at the restoration of homeostasis (Oppenheim, 2001).

Th1 cytokines, IFN-γ and IL-12, play critical roles in fighting against viral infections (Renukaradhya et al., 2010). However, PRRSV infections induce significant suppression of the NK cell cytotoxic activity (Renukaradhya et al., 2010; Dwivedi et al., 2011), leading to delayed and reduced humoral and cell-mediated immune responses due to the increased expression of the anti-inflammatory cytokine IL-10 and thus, suppresses the action of proinflammatory cytokines, such as TNF-α and IFN-γ (Liu et al., 2011). The following have been reported as the roles of TCMs in the modulation of cytokines and inhibition of PRRSV.

### Promoting the Expression IFN-α

Interferon alpha is a type I pleiotropic cytokine that directly participates in several critical host innate and adaptive immune responses. Its ability to promote the production of various potent antiviral mediators that hinder viral replication and packaging makes it very vital in the host defense system (Brockmeier et al., 2012). Also, IFN-α enhances the ability of antigen-presenting cells to activate lymphocytes through the production of cytokines and expression of antigen-presentation molecules. Besides, IFN-α can work directly on T cells to drive maturation from a naïve T cell to an effector cell (Huber and Farrar, 2011). IFN-α is primarily produced in response to a viral infection. Still, it is generally known that PRRSV does not induce a robust IFN-α response, which may secondarily affect the development of an adaptive immune response. Available evidence shows that IFN-α, either as a recombinant protein or through *in vivo* expression of the cloned gene, may enhance the host response to PRRSV, aiding in clearance and increased adaptive immunity (Brockmeier et al., 2012).

*Forsythia suspense* Vahl (Oleaceae) is a widely known TCM herb. The fruit of *F. suspense* is known to contain Forsythoside A, an important bioactive compound with known biological activities against inflammation, pyrexia, sepsis, bacterial (Jiang et al., 2010a, b, 2012), and viral infections (Li H. et al., 2015). It inhibited the replication cycle of PRRSV by significantly reducing the viral RNA copies at the intracellular level in a dose-dependent manner. The suggested mechanism of action is its ability to induce the production of IFN-α actively. This proinflammatory cytokine helps to boost innate immunity in infected MARC-145 cells (Yang M. et al., 2015).

### TCM Impaired the Assembly PRRSV Progeny by Inducing Type 1 IFNs

Dipotassium glycyrrhetate inhibited the proliferation of PRRSV N-protein infection by promoting the production of IFN-α, IFN-β, and IL-1β in PAMs This resulted in the loss of cellular functions in PRRSV and thus, inhibited the assembly of PRRSV progeny in treated cells (Wang et al., 2013).

### Inhibition of Proinflammatory Cytokines

Several authors have reported the anti-inflammatory effects of TCM through modulation of the expression of pro-inflammatory cytokines and chemokines (Mukherjee et al., 2014; Huang et al., 2017) including TNF-α (Fujiki et al., 2003). A study by Ge et al. (2018) showed that increased expression of mRNA transcripts encoding TNF-α, IL-6, and IL-8 after PRRSV infection was reversed by treatment with (–)-epigallocatechin-3-gallate (EGCG). However, treating PRRSV infected cells with EGCG could only suppress viral replication during the early stages of viral infection. The investigation of Zhang et al. (2018b) confirmed that PRRSV infection induced significant RNA expression of three pro-inflammatory cytokines TNF-α, IL-1β, and IL-6, and a relatively small increase in the RNA expression level of IFN-α. It was also observed that the treatment of infected cells with proanthocyanidin A2 (PA2) significantly inhibited PRRSV-induced increase of RNA expression of TNF-α, IL-1β, and IL-6 in PAMs. This was in tandem with earlier reports (Ahmad et al., 2014). They observed that grape seed proanthocyanidin extracts protected mice against carrageenan-induced lung inflammation through the down-regulation of pro-inflammatory cytokines (TNF-α, IL-1β, IL-6, and IFN-γ) and chemokine MCP-1 and inhibition of infiltration of inflammatory cells to the damaged area. It was also reported that proanthocyanidin prevented lipopolysaccharide-induced depression-like behavior in mice through the neuroinflammatory pathway (Jiang et al., 2017). They further observed that proanthocyanidin caused the inhibition of LPS-induced iNOS and COX-2 overexpression by modulating NF-κB pathway and thus inhibited the overexpression of pro-inflammatory cytokines (TNF-α, IL-1β, and IL-6) in the hippocampus.

### Deactivation of IL-1β Secretion and Inhibition of MyD88/NF-κB Signaling Pathway and NLRP3 Inflammasome

Porcine reproductive and respiratory syndrome virus infection triggers the secretion of IL-1β, an essential proinflammatory cytokine synthesized mainly by macrophages, monocytes and dendritic cells that plays a critical role in coordinating the inflammatory and immune responses of the host system against invading pathogens (Eder, 2009). The up-regulation of IL-1β in PRRSV infected PAMs is mediated by the TLR4/MyD88 pathway and NLRP3 inflammasome (Bi et al., 2014). The expression of the TLR4/MyD88 signaling pathway activates the NF-κB pathway. The activation of the NF-κB pathway significantly increases the production of various proinflammatory cytokines, including IL-1β, IL-6, IL-8, and TNF-α, leading to increased pathogenesis and apoptosis. Matrine significantly inhibited the secretion of IL-1β and other proinflammatory cytokines by deactivating the synthesis of MyD88/NF-κB andNLRP3 inflammasome *in vitro* (Sun et al., 2019).

The investigation of Ge and co-workers showed that (–)-epigallocatechin-3-gallate (EGCG) significantly down-regulated proinflammatory cytokines TNF-α, IL-6, and IL-8. Besides, there is a remarkable increase in the production of cytokines including TNF-α, IL-6, and IL-8 especially during the early stage of PRRSV infection (Van Reeth et al., 2002; Thanawongnuwech et al., 2004; Chen et al., 2014). Therefore, the down-regulation of these proinflammatory cytokines and chemokine by treatment with ECGC in PRRSV infected cells was because ECGC was found to reverse the increased expression of mRNA transcripts encoding TNF-α, IL-6 and IL-8 after PRRSV infection and thereby inhibit viral replication and propagation during the early stages of viral infection (Ge et al., 2018). *Sasa quelpaertensis* Nakai extracts have also been reported to significantly reduce the overexpression of proinflammatory cytokines including IL-1a, IL-6, IL-8, IL-15, and TNF-α and synergistically induced the expression of chemokines including IRFs, TLRs, and genes involved in the antiviral immune responses in SQE-treated PRRSV-infected cells. The intervention of SQE disrupted the mechanism with which PRRSV escapes the host innate defense system through the expression of immune-related genes which facilitated the suppression of PRRSV replication *in vitro* (Kang and Lee, 2015).

### Down-Regulation IL-10 by Suppressing N Protein

The pleiotropic IL-10 is an essential cytokine involved in the persistence of viral infection. The expression of IL-10 is up-regulated by N protein which is dependent on the activation of NF-κB. Sodium tanshinone IIA sulfonate (STS) down-regulated IL-10 cytokine expression through the suppression of N protein. STS was observed to attenuate the persistence of PRRSV by down-regulating the expression of IL-10 as a result of the inhibition of transcription and translation of PRRSV N protein which concomitantly led to reduced cell apoptosis (Sun et al., 2014a).

### Deactivation of the NF-κB Pathway

It was also observed that Proanthocyanidin A2 (PA2) inhibited the release of PRRSV progeny particles by blocking the NF-κB pathway. It is present as a dimer of proanthocyanidins in plants tissues. It is produced from the condensation of catechins (Fine, 2000). PA2 and its derivatives exhibit antiviral activities against HSV, Coxsackie B virus (CBV), and CDV (Gallina et al., 2011), by multiple mechanisms depending on the strain of the virus. PA2 inhibited PPRSV infections *in vitro* and targeted the various stages of PPRSV infection including the viral entry, internalization, viral packaging and progeny release phases of the life cycle of PRRSV through numerous mechanisms (Zhang et al., 2018b). PA2 significantly reduced the numbers of PRRSV particles in treated MARC 145 and PAM cells. Zhang et al. (2018b) postulated that the antiviral mechanism of PA2 against PRRSV might be mediated directly through structural alteration or viral inactivation and indirectly through its antioxidant activity. The antioxidant and antidepressant activities of PA2 have been reported (Maldonado et al., 2005; Xu et al., 2010). Interestingly, PRRSV infection is known to induce oxidative stress in cells by generating ROS, thereby leading to the activation of NF-κB pathway, a very significant pathway in PRRSV pathogenesis (Lee and Kleiboeker, 2005). It was therefore inferred that PA2 could indirectly inhibit PRRSV packaging and assembly by inhibiting the activation of NF-κB biosynthesis. However, this needs to be investigated further. See Table 4 for the summary.

**TABLE 4:** Immunomodulators and PRRSV pathogenic pathways inhibitors.

TCMs	Compounds	Antiviral strategy	Mechanism	References
*Forsythia suspense* Vahl	Forsythoside A	Inhibits the replication of PRRSV Reduces the viral RNA copies at the intracellular level	Strongly induces the production of IFN-α in a dose-dependent manner	Yang M. et al., 2015
*Sophora flavescens Ait*	Matrine	Significantly inhibited the secretion of IL-1β	Abrogated the NF-κB signaling pathway by deactivating the expression of MyD88 and NLRP3 inflammasome	Sun et al., 2019
Green Tea (*Camellia sinensis*)	(–)-epigallocatechin-3-gallate (EGCG)	Inhibits early stage viral replication	Reverses the increased expression of mRNA transcripts encoding TNF-α, IL-6, and IL-7	Ge et al., 2018
*Salvia miltiorrhiza*	Tanshinone IIA sulfonate (STS)	Inhibits PRRSV replication	Down-regulates IL-10 cytokine expression through the suppression of N protein	Sun et al., 2012
Licorice roots (*Glycyrrhiza glabra*)	Dipotassium glycyrrhetate	Inhibits PRRSV replication Impairs viral assembly	Inhibits the expression of N protein by modulating the expression of IFNs	Wang et al., 2013
Rhizomes of *Dryopteris crassirhizoma* Nakai	Flavaspidic Acid Ab (Fa-Ab)	Inhibits PRRSV replication	Induces the expression of IFN-α, IFN-β, and interleukin-1β	Yang et al., 2013
Grape seed *Vitis vinifera*	Proanthocyanidin A2 (PA2)	Multiple Strategies: Blocks viral attachment, internalization, viral packaging, and progeny release	Inhibits the release of PRRSV progeny particles by deactivating the NF-κB pathway	Zhang et al., 2018b

## Conclusion

One of the most ubiquitous and notorious viral diseases that pose a perennial threat to the survival of the global swine industry is PRRS. The current attempts to control the disease, including the application of vaccines and veterinary medicine have not achieved the expected result, and the reliable control of PRRSV is presently beyond our reach. The pathway toward PRRS control and the economic importance of swine for human life drive the search for novel, innovative, and alternative strategies for the effective control of the disease, and TCM has proven to have a promising potential in bridging this gap (Montaner-Tarbes et al., 2019).

The antiviral strategies of TCMs are due to their bioactive ingredients. The pathway forward must be built on a real understanding of the mechanisms by which TCMs exerts their effects. Studies focused on the antiviral effect of TCMs against PRRSV have shown that the bioactive ingredients have both direct and indirect inhibition effects on the viral life cycle, including the entry blockers, replication inhibitors, packaging and assembly inhibitors, immunomodulators and pathogenic pathways inhibitors (Ge et al., 2018; Sun et al., 2018; Zhang et al., 2018a, b). These observations raise questions that must be considered in making plans for further research, i.e., the specific mechanism of interaction between the bioactive compounds in herbal extracts and PRRSV virulent proteins; the standard procedures to support the efficacy of available TCMs especially through controlled laboratory and clinical trials; and harness the active bioactive components in TCM materials through combined intervention to synergistically achieve significant effects at extremely low doses. Even more intriguing is on-going research based on advanced techniques and the collaborations in the areas of bioinformatics, chemistry, and proteomics could lead to the availability of sufficient data to support the efficacy, safety, and quality control levels of TCMs without any breach of data integrity and good manufacturing practices.

This review has provided a summary of some TCMs with observed biological activities against the different stages of the life cycle of PRRSV. Two major categories of TCMs were outlined based on their observed mode of actions, including those that directly target a specific stage in the life cycle of the virus (direct-acting antivirals -DAAs) such as entry blockers, replication inhibitors, packaging and assembly inhibitors, and those that indirectly inhibit the growth of the virus through the modification of the host immune system (indirect-acting antivirals -IAAs) such as immunomodulators and inhibitors of metabolic pathways that promote PRRSV pathogenesis.

## Author Contributions

YL and XC designed the structure and concept of the review. GB-O and GW wrote the review. XH, EN, and WC edited the work. FY, YZ, LW, and ZL were also supportive during the writing of the review. All authors have consented and approved the final version of the manuscript.

## Conflict of Interest

The authors declare that the research was conducted in the absence of any commercial or financial relationships that could be construed as a potential conflict of interest.

## Footnotes

1 https://talk.ictvonline.org/taxonomy

## Abbreviations

CD163: cluster of differentiation 163
CD169: cluster of differentiation 169
CDV: Canine Distemper Virus
CFDA: China Food and Drug Administration
DC –SIGN: dendritic cell-specific intercellular adhesion molecule-3-grabbing non-integrin/CD209 (cluster of differentiation 209)
EAV: Equine arteritis virus
ERKs/ERK1/2: Extracellular signal–regulated kinases/Extracellular signal–regulated kinases 1/2
FDA: Food and Drug Administration
H5N1: hemagglutinin-5 and neuraminidase-1
HBV: hepatitis B virus
HCV: hepatitis C virus
HIV: human immunodeficiency virus
HS: heparan sulfate
HSV: herpes simplex virus
ICD: International Statistical Classification of Diseases
IFN-γ: interferon gamma
IL: interleukin
INF-α: interferon alpha
INF-β: interferon beta
IPMA: immunoperoxidase monolayer assay
LDV: lactate dehydrogenase-elevating virus
LV: lelystad virus
MARC 145: Meat Animal Research Center-145 Cell line
MHCII-A: major histocompatibility complex class II antigen molecules
MLV: modified live vaccines
MYH9: non-muscle myosin heavy chain 9
NADC 30: National Animal Disease Center 30 PRRSV strain
NF-κB: nuclear factor kappa-light-chain-enhancer of activated B cells
NK: natural killer cells
NNI: non-nucleoside inhibitors
NS5B RdRp: non-structural protein 5B RNA-dependent RNA polymerase
NSP9: non-structural protein 9
p107: retinoblastoma-like protein 1
PAM: porcine alveolar macrophage
PCD163: porcine cluster of differentiation 163
PERK: protein kinase R (PKR)-like endoplasmic reticulum kinase
PRRSV: porcine reproductive and respiratory syndrome virus
RdRp: RNA-dependent RNA polymerase
ROS: reactive oxygen species
SARS: severe acute respiratory syndrome
SARS-CoV: SARS coronavirus
SHFV: simian hemorrhagic fever virus
TCM: Traditional Chinese Medicine
Th1: T helper 1 (cells or cytokines)
TNF-α: tumor necrosis factor alpha
WHO: World health Organization.

## References

[B1] Adiguzel A. Ozer H. Sokmen M. Gulluce M. Baris O. J. (2009). Antimicrobial and antioxidant activity of the essential oil and methanol extract of *Nepeta cataria*. *Pol. J. Microbiol.* 58 69–76. 19469289

[B2] Aggarwal B. B. Kumar A. Bharti A. C. (2003). Anticancer potential of curcumin: preclinical and clinical studies. *Anticancer Res.* 23 363–398. 12680238

[B3] Ahmad A. van Vuuren S. Viljoen A. (2014). Unravelling the complex antimicrobial interactions of essential oils - the case of *Thymus vulgaris* (Thyme). *Molecules* 19 2896–2910. 10.3390/molecules19032896 24662066 PMC6271043

[B4] Albina E. Carrat C. Charley B. (1998). Interferon-alpha response to swine arterivirus (PoAV), the porcine reproductive and respiratory syndrome virus. *J. Interferon Cytokine Res.* 18 485–490. 9712364 10.1089/jir.1998.18.485

[B5] Amer F. A. (2018). Large-scale hepatitis C combating campaigns in Egypt and Georgia; past, current and future challenges. *J. Infect. Dev. Countr.* 12 404–414. 10.3855/jidc.9784 31940291

[B6] Aqeel M. (2017). Indirect acting antivirals; tricking the virus through a pristine approach. *Annals of Life Sciences* 1, 1–6.

[B7] Arun N. Nalini N. (2002). Efficacy of turmeric on blood sugar and polyol pathway in diabetic albino rats. *Plant Foods Hum. Nutr.* 57 41–52. 10.1023/a:1013106527829 11855620

[B8] Baker S. C. Denison M. R. (2008). “Cell biology of nidovirus replication complexes,” in *Nidoviruses*, eds Perlman S. Gallagher T. Snijder E. J. (Washington, DC: ASM Press), 433.

[B9] Balasch M. Fort M. Taylor L. P. Díaz I. Mateu E. Calvert J. G. (2019). Immune response development after vaccination of 1-day-old naïve pigs with a porcine reproductive and respiratory syndrome 1-based modified live virus vaccine. *Porc. Health Manag.* 5:2. 10.1186/s40813-018-0112-7 30761215 PMC6359793

[B10] Ban S. Ueda Y. Ohashi M. Matsuno K. Ikeda M. Kato N. (2013). Peroxisome proliferator-activated receptor delta antagonists inhibit hepatitis C virus RNA replication. *Bioorg. Med. Chem. Lett.* 23 4774–4778. 10.1016/j.bmcl.2013.07.005 23891183

[B11] Bi J. Song S. Fang L. Wang D. Jing H. Gao L. (2014). Porcine reproductive and respiratory syndrome virus induces IL-1[beta] production depending on TLR4/MyD88 pathway and NLRP3 inflammasome in primary porcine alveolar macrophages. *Hepatology* 2014:403515. 10.1155/2014/403515 24966466 PMC4055429

[B12] Blach S. Zeuzem S. Manns M. Altraif I. Duberg A. S. Muljono D. H. (2017). Global prevalence and genotype distribution of hepatitis C virus infection in 2015: a modelling study. *Lancet Gastroenterol. Hepatol.* 2 161–176. 10.1016/s2468-1253(16)30181-9 28404132

[B13] Boon J. A. D. Spaan W. J. M. Virology E. J. S. J. (1995). Equine arteritis virus subgenomic RNA transcription: UV inactivation and translation inhibition studies. *Cell* 213:364. 10.1006/viro.1995.0009 PMC7131190 7491761

[B14] Brockmeier S. L. Loving C. L. Eberle K. C. Hau S. J. Buckley A. Van Geelen A. (2017). Interferon alpha inhibits replication of a live-attenuated porcine reproductive and respiratory syndrome virus vaccine preventing development of an adaptive immune response in swine. *Microbiology* 212 48–51. 10.1016/j.vetmic.2017.11.004 29173587

[B15] Brockmeier S. L. Loving C. L. Nelson E. A. Miller L. C. Nicholson T. L. Register K. B. (2012). The presence of alpha interferon at the time of infection alters the innate and adaptive immune responses to porcine reproductive and respiratory syndrome virus. *Clin. Vaccine Immunol.* 19 508–514. 10.1128/cvi.05490-11 22301694 PMC3318278

[B16] Burns J. J. Zhao L. Taylor E. W. Spelman K. (2010). The influence of traditional herbal formulas on cytokine activity. *Toxicology* 278 140–159. 10.1016/j.tox.2009.09.020 19818374

[B17] Carrillo J. L. M. Rodríguez F. P. C. Coronado O. G. García M. A. M. Cordero J. F. C. (2017). “Physiology and pathology of innate immune response against pathogens,” in *Physiology and Pathology of Immunology*, ed. Rezaei N. (IntechOpen). 10.5772/intechopen.70556

[B18] Chacko S. M. Thambi P. T. Kuttan R. Nishigaki I. J. (2010). Beneficial effects of green tea: a literature review. *Chin. Med.* 5:13. 10.1186/1749-8546-5-13 20370896 PMC2855614

[B19] Charerntantanakul W. (2012). Porcine reproductive and respiratory syndrome virus vaccines: immunogenicity, efficacy and safety aspects. *World J. Virol.* 1 23–30. 24175208 10.5501/wjv.v1.i1.23 PMC3782261

[B20] Chen K. Yu B. (1999). Certain progress of clinical research on Chinese integrative medicine. *Chin. Med. J.* 112 934–937. 11717980

[B21] Chen M. Z. Xie H. Yang L. W. Liao Z. H. Yu J. (2010). In vitro anti-influenza virus activities of sulfated polysaccharide fractions from *Gracilaria lemaneiformis*. *Virol. Sin.* 25:341351. 10.1007/s12250-010-3137-x PMC8227872 20960180

[B22] Chen X. X. Quan R. Guo X. K. Gao L. Shi J. Feng W. H. (2014). Up-regulation of pro-inflammatory factors by HP-PRRSV infection in microglia: implications for HP-PRRSV neuropathogenesis. *Vet. Microbiol.* 170 48–57. 10.1016/j.vetmic.2014.01.031 24581811

[B23] Cheng J. S. N. Zhao X. Niu L. Song M. Sun Y. Jiang J. (2013). In vitro screening for compounds derived from traditional chinese medicines with antiviral activities against porcine reproductive and respiratory syndrome virus. *J. Microbiol. Biotechnol.* 23 1076–1083. 10.4014/jmb.1303.0307 23727804

[B24] Christman J. W. Sadikot R. T. Blackwell T. S. (2000). The role of nuclear factor-kappa B in pulmonary diseases. *Chest* 117 1482–1487. 10.1378/chest.117.5.1482 10807839

[B25] Cinatl J. Morgenstern B. Bauer G. Chandra P. Rabenau H. Doerr H. W. (2003). Glycyrrhizin, an active component of liquorice roots, and replication of SARS-associated coronavirus. *Lancet* 361 2045–2046. 10.1016/s0140-6736(03)13615-x 12814717 PMC7112442

[B26] Colpitts C. C. Schang L. M. (2014). A small molecule inhibits virion attachment to heparan sulfate- or sialic acid-containing glycans. *J. Virol.* 88 7806–7817. 10.1128/JVI.00896-14 24789779 PMC4097786

[B27] Darrick K. L. Chung R. T. (2019). Overview of direct-acting antiviral drugs and drug resistance of hepatitis C virus. *Methods Mol. Biol.* 191 3–32. 10.1007/978-1-4939-8976-8_1 30593615

[B28] Darwich L. Díaz I. Mateu E. (2010). Certainties, doubts and hypotheses in porcine reproductive and respiratory syndrome virus immunobiology. *Virus Res.* 154 123–132. 10.1016/j.virusres.2010.07.017 20659507

[B29] Dhingra S. Sharma A. K. Arora R. C. Slezak J. Singal P. K. (2009). IL-10 attenuates TNF-alpha-induced NF kappa B pathway activation and cardiomyocyte apoptosis. *Cardiovasc. Res.* 82 59–66. 10.1093/cvr/cvp040 19181934

[B30] Diaz I. Darwich L. Pappaterra G. Pujols J. Mateu E. (2006). Different European-type vaccines against porcine reproductive and respiratory syndrome virus have different immunological properties and confer different protection to pigs. *Virology* 351 249–259. 10.1016/j.virol.2006.03.046 16712895

[B31] DiDonato J. A. Hayakawa M. Rothwarf D. M. Zandi E. Karin M. (1997). A cytokine-responsive IkappaB kinase that activates the transcription factor NF-kappaB. *Nature* 388 548–554. 10.1038/41493 9252186

[B32] Dong J. Zhang N. Ge X. Zhou L. Guo X. Yang H. (2014). The interaction of nonstructural protein 9 with retinoblastoma protein benefits the replication of genotype 2 porcine reproductive and respiratory syndrome virus in vitro. *Virology* 464–465 432–440. 10.1016/j.virol.2014.07.036 25146601 PMC7112046

[B33] Du T. Nan Y. Xiao S. Zhao Q. Zhou E. M. (2017a). Antiviral strategies against PRRSV infection. *Trends Microbiol.* 25 968–979. 10.1016/j.tim.2017.06.001 28652073

[B34] Du T. Shi Y. Xiao S. Li N. Zhao Q. Zhang A. (2017b). Curcumin is a promising inhibitor of genotype 2 porcine reproductive and respiratory syndrome virus infection. *BMC Vet. Res.* 13:298. 10.1186/s12917-017-1218-x 29017487 PMC5633875

[B35] Duan E. Wang D. Fang L. Ma J. Luo J. Chen H. (2015). Suppression of porcine reproductive and respiratory syndrome virus proliferation by glycyrrhizin. *Antiviral Res.* 120 122–125. 10.1016/j.antiviral.2015.06.001 26055123 PMC7113688

[B36] Dwivedi V. Manickam C. Patterson R. Dodson K. Murtaugh M. Torrelles J. B. (2011). Cross-protective immunity to porcine reproductive and respiratory syndrome virus by intranasal delivery of a live virus vaccine with a potent adjuvant. *Vaccine* 29 4058–4066. 10.1016/j.vaccine.2011.03.006 21419162 PMC7127856

[B37] Ebrahimi R. Zamani Z. Kashi A. (2009). Genetic diversity evaluation of wild Persian shallot (*Allium hirtifolium* Boiss.) using morphological and RAPD markers. *Sci. Hortic.* 119 345–351. 10.1016/j.scienta.2008.08.032

[B38] Eder C. J. I. (2009). Mechanisms of interleukin-1β release. *Immunobiology* 214 543–553. 10.1016/j.imbio.2008.11.007 19250700

[B39] Faaberg K. S. (2008). “Arterivirus structural proteins and assembly,” in *Nidoviruses*, eds Perlman S. Gallagher T. Snijder E. J. (Washington, DC: ASM Press), 433.

[B40] Faisst S. (1999). Propagation of viruses. *Anim. Encyclop. Virol.* 44 1408–1413.

[B41] Fine A. M. (2000). Oligomeric proanthocyanidin complexes: history, structure, and phytopharmaceutical applications. *Altern. Med. Rev.* 5 144–151. 10767669

[B42] Friedman M. (2007). Overview of antibacterial, antitoxin, antiviral, and antifungal activities of tea flavonoids and teas. *Mol. Nutr. Food Res.* 51 116–134. 10.1002/mnfr.200600173 17195249 PMC7168386

[B43] Fujiki H. Suganuma M. Kurusu M. Okabe S. Imayoshi Y. Taniguchi S. (2003). New TNF-alpha releasing inhibitors as cancer preventive agents from traditional herbal medicine and combination cancer prevention study with EGCG and sulindac or tamoxifen. *Mutat. Res.* 523–524 119–125. 10.1016/s0027-5107(02)00327-5 12628509

[B44] Gallina L. Dal Pozzo F. Galligioni V. Bombardelli E. Scagliarini A. (2011). Inhibition of viral RNA synthesis in canine distemper virus infection by proanthocyanidin A2. *Antiviral Res.* 92 447–452. 10.1016/j.antiviral.2011.10.004 22020306

[B45] Gao J. Xiao S. Xiao Y. Wang X. Zhang C. Zhao Q. (2016). MYH9 is an essential factor for porcine reproductive and respiratory syndrome virus infection. *Sci. Rep.* 6:25120. 10.1038/srep25120 27112594 PMC4845007

[B46] Gao L. Zhang W. Sun Y. Yang Q. Ren J. Liu J. (2013). *Cryptoporus volvatus* extract inhibits porcine reproductive and respiratory syndrome virus (PRRSV) in vitro and in vivo. *Cell* 8:e63767. 10.1371/journal.pone.0063767 PMC3660591 23704937

[B47] Garjani A. Afrooziyan A. Nazemiyeh H. Najafi M. Kharazmkia A. Maleki-Dizaji N. (2009). Protective effects of hydroalcoholic extract from rhizomes of *Cynodon dactylon* (L.) Pers. on compensated right heart failure in rats. *BMC Complement. Altern. Med.* 9:28. 10.1186/1472-6882-9-28 19653918 PMC2734535

[B48] Garozzo A. Timpanaro R. Stivala A. Bisignano G. Castro A. (2011). Activity of *Melaleuca alternifolia* (tea tree) oil on Influenza virus A/PR/8: study on the mechanism of action. *Antiviral Res.* 89 83–88. 10.1016/j.antiviral.2010.11.010 21095205

[B49] Gastaminza P. Whitten-Bauer C. Chisari F. V. (2010). Unbiased probing of the entire hepatitis C virus life cycle identifies clinical compounds that target multiple aspects of the infection. *Proc. Natl. Acad. Sci. U.S.A.* 107 291–296. 10.1073/pnas.0912966107 19995961 PMC2806752

[B50] Ge M. Xiao Y. Chen H. Luo F. Du G. Zeng F. (2018). Multiple antiviral approaches of (-)-epigallocatechin-3-gallate (EGCG) against porcine reproductive and respiratory syndrome virus infection in vitro. *Antiviral Res.* 158 52–62. 10.1016/j.antiviral.2018.07.012 30048655

[B51] Gordon C. J. Tchesnokov E. P. Feng J. Y. Porter D. P. Götte M. (2020a). The antiviral compound remdesivir potently inhibits RNA-dependent RNA polymerase from Middle East respiratory syndrome coronavirus. *J. Biol. Chem.* 295 4773–4779. 10.1074/jbc.AC120.013056 32094225 PMC7152756

[B52] Gordon C. J. Tchesnokov E. P. Woolner E. Perry J. K. Feng J. Y. Porter D. P. (2020b). Remdesivir is a direct-acting antiviral that inhibits RNA-dependent RNA polymerase from severe acute respiratory syndrome coronavirus 2 with high potency. *J. Biol. Chem.* 295 6785–6797. 10.1074/jbc.RA120.013679 32284326 PMC7242698

[B53] Guo C. Zhu Z. Wang X. Chen Y. Liu X. J. V. M. (2017). Pyrithione inhibits porcine reproductive and respiratory syndrome virus replication through interfering with NF-κB and heparanase. *Vet. Microbiol.* 201 231–239. 10.1016/j.vetmic.2017.01.033 28284615

[B54] Gupta P. Kumar R. Garg P. Singh I. P. (2010). Active site binding modes of dimeric phloroglucinols for HIV-1 reverse transcriptase, protease and integrase. *Bioorg. Med. Chem. Lett.* 20 4427–4431. 10.1016/j.bmcl.2010.06.057 20594846

[B55] Hadigal S. R. Agelidis A. M. Karasneh G. A. Antoine T. E. Yakoub A. M. Ramani V. C. (2015). Heparanase is a host enzyme required for herpes simplex virus-1 release from cells. *Nat. Commun.* 6:6985. 10.1038/ncomms7985 25912399 PMC4413471

[B56] Holmes J. A. Rutledge S. M. Chung R. T. (2019). Direct-acting antiviral treatment for hepatitis C. *Lancet* 393:10179. 10.1016/S0140-6736(18)32326-2 30765125

[B57] Huang C. Zhang Q. Guo X. K. Yu Z. B. Xu A. T. Tang J. (2014). Porcine reproductive and respiratory syndrome virus nonstructural protein 4 antagonizes beta interferon expression by targeting the NF-κB essential modulator. *J. Virol.* 88 10934–10945. 10.1128/JVI.01396-14 25008936 PMC4178863

[B58] Huang F. Ding J. Miao L. Zhao Y. X. Zhang M. Y. Chen C. (2017). Effects of EGCG on LPS-induced elevation of inflammatory factors in human gingival fibroblasts and functional mechanisms. *Int. J. Clin. Exp. Pathol.* 10 5580–5586.

[B59] Huang W. Chen X. Li Q. Li P. Zhao G. Xu M. (2012). Inhibition of intercellular adhesion in herpex simplex virus infection by glycyrrhizin. *Cell Biochem. Biophys.* 62 137–140. 10.1007/s12013-011-9271-8 21874590

[B60] Huber J. P. Farrar J. D. (2011). Regulation of effector and memory T-cell functions by type I interferon. *Immunology* 132 466–474. 10.1111/j.1365-2567.2011.03412.x 21320124 PMC3075500

[B61] Immanuel G. Uma R. P. Iyapparaj P. Citarasu T. Peter S. M. P. Babu M. M. (2009). Dietary medicinal plant extracts improve growth, immune activity and survival of tilapia *Oreochromis mossambicus*. *J. Fish Biol.* 74 1462–1475. 10.1111/j.1095-8649.2009.02212.x 20735646

[B62] Jiang W. L. Fu F. H. Xu B. M. Tian J. W. Zhu H. B. Hou J. (2010a). Cardioprotection with forsythoside B in rat myocardial ischemia-reperfusion injury: relation to inflammation response. *Phytomedicine* 17 635–639. 10.1016/j.phymed.2009.10.017 19959348

[B63] Jiang W. L. Tian J. W. Fu F. H. Zhu H. B. Hou J. (2010b). Neuroprotective efficacy and therapeutic window of Forsythoside B: in a rat model of cerebral ischemia and reperfusion injury. *Eur. J. Pharmacol.* 640 75–81. 10.1016/j.ejphar.2010.04.055 20470770

[B64] Jiang W. L. Yong X. Zhang S. P. Zhu H. B. Hou J. (2012). Forsythoside B protects against experimental sepsis by modulating inflammatory factors. *Phytother. Res.* 26 981–987. 10.1002/ptr.3668 22147417

[B65] Jiang X. Liu J. Lin Q. Mao K. Tian F. Jing C. (2017). Proanthocyanidin prevents lipopolysaccharide-induced depressive-like behavior in mice via neuroinflammatory pathway. *Brain Res. Bull.* 135 40–46. 10.1016/j.brainresbull.2017.09.010 28941603

[B66] Jiang Z. Zhou E. M. Ameri-Mahabadi M. Zimmerman J. J. Platt K. B. (2003). Identification and characterization of auto-anti-idiotypic antibodies specific for antibodies against porcine reproductive and respiratory syndrome virus envelope glycoprotein (GP5). *Vet. Immunol. Immunopathol.* 92 125–135. 10.1016/s0165-2427(03)00022-9 12730013

[B67] Kaewprom K. Chen Y.-H. Lin C.-F. Chiou M.-T. Lin C.-N. (2017). Antiviral activity of *Thymus vulgaris* and *Nepeta cataria* hydrosols against porcine reproductive and respiratory syndrome virus. *Thai J. Vet. Med.* 47 25–33.

[B68] Kang H. Lee C. (2015). Sasa quelpaertensisNakai extract suppresses porcine reproductive and respiratory syndrome virus replication and modulates virus-induced cytokine production. *Arch. Virol.* 160 1977–1988. 10.1007/s00705-015-2469-0 26047649 PMC7087003

[B69] Kapadia G. J. Tokuda H. Konoshima T. Takasaki M. Takayasu J. Nishino H. (1996). Anti-tumor promoting activity of *Dryopteris phlorophenone* derivatives. *Cancer Lett* 105 161–165. 10.1016/0304-3835(96)04275-9 8697439

[B70] Kappes M. A. Faaberg K. S. (2015). PRRSV structure, replication and recombination: origin of phenotype and genotype diversity. *Virology* 479–480 475–486. 10.1016/j.virol.2015.02.012 25759097 PMC7111637

[B71] Kielian M. Jungerwirth S. (1990). Mechanisms of enveloped virus entry into cells. *Biophys. J.* 7:17. 2182968

[B72] Kim H. Y. Park E. J. Joe E. H. Jou I. (2003). Curcumin suppresses janus kinase-STAT inflammatory signaling through activation of Src homology 2 domain-containing tyrosine phosphatase 2 in brain microglia. *J. Immunol.* 171 6072–6079. 10.4049/jimmunol.171.11.6072 14634121

[B73] Kim J. K. Fahad A. M. Shanmukhappa K. Kapil S. (2006). Defining the cellular target(s) of porcine reproductive and respiratory syndrome virus blocking monoclonal antibody 7G10. *J. Virol.* 80 689–696. 10.1128/JVI.80.2.689-696.2006 16378972 PMC1346842

[B74] Kimman T. G. (1992). Risks connected with the use of conventional and genetically engineered vaccines. *Vet. Q.* 14 110–118. 10.1080/01652176.1992.9694344 1413441

[B75] Klasse P. J. Bron R. Marsh M. (1998). Mechanism of enveloped virus entry into animal cells. *Mol. Biol. Med.* 34 65–91. 10.1016/S0169-409X(98)00002-7 PMC7125647 10837671

[B76] Koyama S. Ishii K. J. Coban C. Akira S. (2008). Innate immune response to viral infection. *Cytokine* 43 336–341. 10.1016/j.cyto.2008.07.009 18694646

[B77] Kreutz L. C. Ackermann M. R. (1996). Porcine reproductive and respiratory syndrome virus enters cells through a low pH-dependent endocytic pathway. *Virus Res.* 42 137–147. 8806181 10.1016/0168-1702(96)01313-5

[B78] Kuete V. Sandjo L. P. (2012). Isobavachalcone: an overview. *Chin. J. Integr. Med.* 18 543–547. 10.1007/s11655-012-1142-7 22772918

[B79] Kuo P.-C. Lin T.-C. Yang C.-W. Lin C.-L. Chen G.-F. Huang J.-W. (2010). Bioactive saponin from tea seed pomace with inhibitory effects against *Rhizoctonia solani*. *J. Agric. Food Chem.* 58 8618–8622. 10.1021/jf1017115 20681650

[B80] Lee S. M. Kleiboeker S. B. (2005). Porcine arterivirus activates the NF-kappa B pathway through I kappa B degradation. *Virology* 342 47–59. 10.1016/j.virol.2005.07.034 16129468 PMC7111765

[B81] Lee S.-M. Na M.-K. An R.-B. Min B.-S. Lee H.-K. (2003). Antioxidant activity of two phloroglucinol derivatives from *Dryopteris crassirhizoma*. *Biol. Pharm. Bull.* 26 1354–1356. 10.1248/bpb.26.1354 12951487

[B82] Lehmann K. C. Gorbalenya A. E. Snijder E. J. Posthuma C. C. (2016). Arterivirus RNA-dependent RNA polymerase: vital enzymatic activity remains elusive. *Virology* 487 68–74. 10.1016/j.virol.2015.10.002 26499043 PMC7111705

[B83] Lei Z. Jie R. Peidian S. Dong L. Chengxue Z. Yanxin S. (2018). The immunological regulation roles of porcine β-1, 4 galactosyltransferase V (B4GALT5) in PRRSV infection. *Front. Cell Infect. Microbiol.* 8:48. 10.3389/fcimb.2018.00048 29546034 PMC5837993

[B84] Lei Z. Yang H. J. V. R. (2010). Porcine reproductive and respiratory syndrome in China. *Virus Res.* 154 31–37. 10.1016/j.virusres.2010.07.016 20659506

[B85] Li E. Sun N. Zhao J.-X. Sun Y.-G. Huang J.-G. Lei H.-M. (2015). In vitro evaluation of antiviral activity of tea seed saponins against porcine reproductive and respiratory syndrome virus. *Antiviral Ther.* 20 743–752. 10.3851/imp2937 25609356

[B86] Li H. Wu J. Zhang Z. Ma Y. Liao F. Zhang Y. (2015). Forsythoside A inhibits the avian infectious bronchitis virus in cell culture. *Phytother. Res.* 25 338–342. 10.1002/ptr.3260 20677175 PMC7168103

[B87] Li J. Guo D. Huang L. Yin M. Liu Q. Wang Y. (2014). The interaction between host Annexin A2 and viral Nsp9 is beneficial for replication of porcine reproductive and respiratory syndrome virus. *Virus Res.* 189 106–113. 10.1016/j.virusres.2014.05.015 24874199

[B88] Li T. Peng T. (2013). Traditional Chinese herbal medicine as a source of molecules with antiviral activity. *Antiviral Res.* 97 1–9. 10.1016/j.antiviral.2012.10.006 23153834 PMC7114103

[B89] Lim S. P. Noble C. G. Nilar S. Shi P.-Y. Yokokawa F. (2018). Discovery of potent non-nucleoside inhibitors of dengue viral RNA-dependent RNA polymerase from fragment screening and structure-guided design. dengue and zika: control and antiviral treatment strategies. *Adv. Exp. Med. Biol.* 1062 187–198. 10.1007/978-981-10-8727-1_14 29845534

[B90] Lindenbach B. D. Evans M. J. Syder A. J. Wölk B. Tellinghuisen T. L. Liu C. C. (2005). Complete replication of hepatitis C virus in cell culture. *Science* 309 623–626. 10.1126/science.1114016 15947137

[B91] Liu L. Tian J. Nan H. Tian M. Li Y. Xu X. (2016). Porcine reproductive and respiratory syndrome virus nucleocapsid protein interacts with Nsp9 and cellular DHX9 to regulate viral RNA synthesis. *J. Virol.* 90: JVI.03216-15. 10.1128/JVI.03216-15 27009951 PMC4934760

[B92] Liu Y. Wang L. Kikuiri T. Akiyama K. Chen C. Xu X. (2011). Mesenchymal stem cell-based tissue regeneration is governed by recipient T lymphocytes via IFN-γ and TNF-α. *Nat. Med.* 17 1594–1601. 10.1038/nm.2542 22101767 PMC3233650

[B93] Lunney J. K. Fang Y. Ladinig A. Chen N. Li Y. Rowland B. (2016). Porcine reproductive and respiratory syndrome virus (PRRSV): pathogenesis and interaction with the immune system. *Annu. Rev. Anim. Biosci.* 4 129–154. 10.1146/annurev-animal-022114-111025 26646630

[B94] Ma Z. Zhang W. Wang L. Zhu M. Wang H. Feng W.-H. (2013). A novel compound from the mushroom *Cryptoporus volvatus* inhibits porcine reproductive and respiratory syndrome virus (PRRSV) in vitro. *PLoS One* 8:79333. 10.1371/journal.pone.0079333 24260198 PMC3832501

[B95] Maclachlan N. J. Dubovi E. J. Barthold S. W. Swayne D. E. Winton J. R. (2016). *Fenner’s Veterinary Virology*, 5th Edn Amsterdam: Elsevier Inc.

[B96] Mahady G. B. Pendland S. L. Yun G. Lu Z. Z. (2002). Turmeric (*Curcuma longa*) and curcumin inhibit the growth of *Helicobacter* pylori, a group 1 carcinogen. *Anticancer Res.* 22 4179–4181. 12553052

[B97] Maldonado P. D. Rivero-Cruz I. Mata R. Pedraza-Chaverrí J. (2005). Antioxidant activity of A-type proanthocyanidins from *Geranium niveum* (*Geraniaceae*). *J. Agric. Food Chem.* 53 1996–2001. 10.1021/jf0483725 15769126

[B98] Mardassi H. Massie B. Dea S. (1996). Intracellular synthesis, processing, and transport of proteins encoded by ORFs 5 to 7 of porcine reproductive and respiratory syndrome virus. *Virology* 221 98–112. 10.1006/viro.1996.0356 8661418

[B99] Mathekga A. D. M. Meyer J. J. M. Horn M. M. Drewes S. E. (2000). An acylated phloroglucinol with antimicrobial properties from *Helichrysum caespititium*. *Phytochemistry* 53 93–96. 10.1016/s0031-9422(99)00424-0 10656414

[B100] Mathew D. Hsu W. L. (2018). Antiviral potential of curcumin. *J. Funct. Foods* 40:699 10.1016/j.jff.2017.12.017

[B101] Matsumoto Y. Matsuura T. Aoyagi H. Matsuda M. Hmwe S. S. Date T. (2013). Antiviral activity of glycyrrhizin against hepatitis c virus in vitro. *PLoS One* 8:68992. 10.1371/journal.pone.0068992 23874843 PMC3715454

[B102] Meier W. A. Galeota J. Osorio F. A. Husmann R. J. Schnitzlein W. M. Zuckermann F. A. (2003). Gradual development of the interferon-gamma response of swine to porcine reproductive and respiratory syndrome virus infection or vaccination. *Virology* 309 18–31. 10.1016/s0042-6822(03)00009-6 12726723

[B103] Meng X. J. (2000). Heterogeneity of porcine reproductive and respiratory syndrome virus: implications for current vaccine efficacy and future vaccine development. *Vet. Microbiol.* 74 309–329. 10.1016/s0378-1135(00)00196-6 10831854 PMC7117501

[B104] Meng X. J. Paul P. S. Halbur P. G. Lum M. A. (1995). Phylogenetic analyses of the putative M (ORF 6) and N (ORF 7) genes of porcine reproductive and respiratory syndrome virus (PRRSV): implication for the existence of two genotypes of PRRSV in the U.S.A. and Europe. *Arch. Virol.* 140 745–755. 10.1007/bf01309962 7794115 PMC7086766

[B105] Moghadamtousi S. Z. Kadir H. A. Hassandarvish P. Tajik H. Zandi K. (2014). A review on antibacterial, antiviral, and antifungal activity of curcumin. *Biomed. Res. Intern.* 2014:186864. 10.1155/2014/186864 PMC4022204 24877064

[B106] Molina R. M. Cha S. H. Chittick W. Lawson S. Murtaugh M. P. Nelson E. A. (2008). Immune response against porcine reproductive and respiratory syndrome virus during acute and chronic infection. *Vet. Immunol. Immunopathol.* 126 283–292. 10.1016/j.vetimm.2008.08.002 18835044

[B107] Montaner-Tarbes S. del Portillo H. A. Montoya M. Fraile L. (2019). Key gaps in the knowledge of the porcine respiratory reproductive syndrome virus (PRRSV). *Front. Vet. Sci.* 6:38. 10.3389/fvets.2019.00038 30842948 PMC6391865

[B108] Moore K. W. de Malefyt R. W. Coffman R. L. O’Garra A. (2001). Interleukin-10 and the interleukin-10 receptor. *Annu. Rev. Immunol.* 19 683–765. 10.1146/annurev.immunol.19.1.683 11244051

[B109] Mori T. Miyamoto T. Yoshida H. Asakawa M. Kawasumi M. Kobayashi T. (2011). IL-1β and TNFα-initiated IL-6-STAT3 pathway is critical in mediating inflammatory cytokines and RANKL expression in inflammatory arthritis. *Int. Immunol.* 23 701–712. 10.1093/intimm/dxr077 21937456

[B110] Mukherjee S. Siddiqui M. A. Dayal S. Ayoub Y. Z. Malathi K. (2014). Epigallocatechin-3-gallate suppresses proinflammatory cytokines and chemokines induced by Toll-like receptor 9 agonists in prostate cancer cells. *J. Inflamm. Res.* 7 89–101. 10.2147/JIR.S61365 24971028 PMC4070858

[B111] Nauwynck H. J. Duan X. Favoreel H. W. Van Oostveldt P. Pensaert M. B. (1999). Entry of porcine reproductive and respiratory syndrome virus into porcine alveolar macrophages via receptor-mediated endocytosis. *J. Gen. Virol.* 80(Pt 2), 297–305. 10073688 10.1099/0022-1317-80-2-297

[B112] Nguyen M. D. Julien J.-P. Rivest S. J. N. (2002). Innate immunity: the missing link in neuroprotection and neurodegeneration? *Nat. Rev. Neurosci.* 3 216–227. 10.1038/nrn752 11994753

[B113] Nikolic M. Glamoclija J. Ferreira I. C. F. R. Calhelha R. C. Fernandes A. Markovic T. (2014). Chemical composition, antimicrobial, antioxidant and antitumor activity of *Thymusserpyllum* L., *Thymus algeriensis* Boiss., and Reut, and *Thymus vulgaris* L. essential oils. *Industr. Crops Products* 52 183–190. 10.1016/j.indcrop.2013.10.006

[B114] Oppenheim J. J. (2001). Cytokines: past, present, and future. *Int. J. Hematol.* 74 3–8. 10.1007/BF02982543 11530802

[B115] Pasternak A. O. Spaan W. J. Snijder E. J. (2006). Nidovirus transcription: how to make sense? *J. Gen. Virol.* 87(Pt 6), 1403–1421. 10.1099/vir.0.81611-0 16690906

[B116] Patel D. Nan Y. Shen M. Ritthipichai K. Zhu X. Zhang Y. J. (2010). Porcine reproductive and respiratory syndrome virus inhibits type I interferon signaling by blocking STAT1/STAT2 nuclear translocation. *J. Virol.* 84 11045–11055. 20739522 10.1128/JVI.00655-10 PMC2953160

[B117] Pietschmann T. Kaul A. Koutsoudakis G. Shavinskaya A. Kallis S. Steinmann K. (2006). Construction and characterization of infectious intragenotypic and intergenotypic hepatitis C virus chimeras. *Proc. Natl. Acad. Sci. U.S.A.* 103 7408–7413. 10.1073/pnas.0504877103 16651538 PMC1455439

[B118] Pol J. M. Wagenaar F. Reus J. E. (1997). Comparative morphogenesis of three PRRS virus strains. *Vet. Microbiol.* 55 203–208. 10.1016/s0378-1135(96)01329-6 9220615

[B119] Praditya D. Kirchhoff L. Brüning J. Rachmawati H. Steinmann E. (2019). Anti-infective properties of the golden spice curcumin. *Front. Microbiol.* 10:912. 10.3389/fmicb.2019.00912 31130924 PMC6509173

[B120] Prather R. S. Rowland R. R. Ewen C. Trible B. Kerrigan M. Bawa B. (2013). An intact sialoadhesin (Sn/SIGLEC1/CD169) is not required for attachment/internalization of the porcine reproductive and respiratory syndrome virus. *J. Virol.* 87 9538–9546. 10.1128/JVI.00177-13 23785195 PMC3754101

[B121] Pringproa K. Khonghiran O. Kunanoppadol S. Potha T. Chuammitri P. (2014). In Vitro virucidal and virustatic properties of the crude extract of *Cynodon dactylon* against porcine reproductive and respiratory syndrome virus. *Vet. Med. Intern.* 2014:947589. 10.1155/2014/947589 24744959 PMC3972871

[B122] Qiu M. Chen Y. Chu Y. Song S. Yang N. Gao J. (2013). Zinc ionophores pyrithione inhibits herpes simplex virus replication through interfering with proteasome function and NF-κB activation. *Antiviral Res.* 100 44–53. 10.1016/j.antiviral.2013.07.001 23867132

[B123] Reddy R. C. Vatsala P. G. Keshamouni V. G. Padmanaban G. Rangarajan P. N. (2005). Curcumin for malaria therapy. *Biochem. Biophys. Res. Commun.* 326 472–474. 10.1016/j.bbrc.2004.11.051 15582601

[B124] Renukaradhya G. J. Alekseev K. Jung K. Fang Y. Saif L. J. (2010). Porcine reproductive and respiratory syndrome virus-induced immunosuppression exacerbates the inflammatory response to porcine respiratory coronavirus in pigs. *Viral Immunol.* 23 457–466. 10.1089/vim.2010.0051 20883160 PMC2967820

[B125] Ryu W.-S. (2017). “Antiviral therapy,” in *Molecular Virology of Human Pathogenic Viruses*, 2nd Edn, ed. Ryu W.-S. (San Diego, CA: Academic Press).

[B126] Sagong M. Lee C. (2011). Porcine reproductive and respiratory syndrome virus nucleocapsid protein modulates interferon-β production by inhibiting IRF3 activation in immortalized porcine alveolar macrophages. *Arch. Virol.* 156 2187–2195. 10.1007/s00705-011-1116-7 21947566 PMC7086947

[B127] Samer S. E.-K. Shyamasundaran K. (2020). “Viral hepatitis,” in *Hunter’s Tropical Medicine and Emerging Infectious Diseases*, 10th Edn, eds Ryan E. T. Hill D. R. Aronson N. E. Endy T. P. (Amsterdam: Elsevier), 308–324. 10.1016/C2016-0-01879-X

[B128] Sandle T. (2015). Current methods and approaches for viral clearance. *Am. Pharm. Rev.* 2015 1–4.

[B129] Sato H. Goto W. Yamamura J. Kurokawa M. Kageyama S. Takahara T. (1996). Therapeutic basis of glycyrrhizin on chronic hepatitis B. *Antiviral Res.* 30 171–177. 10.1016/0166-3542(96)00942-4 8783808

[B130] Shi X. Wang L. Li X. Gaiping Z. Guo J. Zhao D. (2011). Endoribonuclease activities of porcine reproductive and respiratory syndrome virus nsp11 was essential for nsp11 to inhibit IFN-beta induction. *Mol. Immunol.* 48 1568–1572. 21481939 10.1016/j.molimm.2011.03.004 PMC7112683

[B131] Singh S. K. Kesari A. N. Gupta R. K. Jaiswal D. Watal G. (2007). Assessment of antidiabetic potential of *Cynodon dactylon* extract in streptozotocin diabetic rats. *J. Ethnopharmacol.* 114 174–179. 10.1016/j.jep.2007.07.039 17889469

[B132] Snijder E. J. (1998). The arterivirus replicase: the road from RNA to protein(s), and back again. *Adv. Exp. Med. Biol.* 440 97–108. 9782270

[B133] Snijder E. J. Meulenberg J. J. (1998). The molecular biology of arteriviruses. *J. Gen. Virol.* 79(Pt 5), 961–979. 10.1099/0022-1317-79-5-961 9603311

[B134] Song S. Bi J. Wang D. Fang L. Zhang L. Li F. (2013). Porcine reproductive and respiratory syndrome virus infection activates IL-10 production through NF-κB and p38 MAPK pathways in porcine alveolar macrophages. *Dev. Comp. Immunol.* 39 265–272. 10.1016/j.dci.2012.10.001 23085400

[B135] Steinmann J. Buer J. Pietschmann T. Steinmann E. (2013). Anti-infective properties of epigallocatechin-3-gallate (EGCG), a component of green tea. *Br. J. Pharmacol.* 168 1059–1073. 10.1111/bph.12009 23072320 PMC3594666

[B136] Su X. Z. Miller L. H. (2015). The discovery of artemisinin and the nobel prize in physiology or medicine. *Sci. China Life Sci.* 58 1175–1179. 10.1007/s11427-015-4948-7 26481135 PMC4966551

[B137] Sun N. Li E. Wang Z. Zhao J. Wang S. He J. (2014a). Sodium tanshinone IIA sulfonate inhibits porcine reproductive and respiratory syndrome virus via suppressing N gene expression and blocking virus-induced apoptosis. *Antivir. Ther.* 19 89–95. 10.3851/imp2694 24158620

[B138] Sun N. Wang Z.-W. Wu C.-H. Li E. He J.-P. Wang S.-Y. (2014b). Antiviral activity and underlying molecular mechanisms of matrine against porcine reproductive and respiratory syndrome virus in vitro. *Science* 96 323–327. 10.1016/j.rvsc.2013.12.009 24411654

[B139] Sun N. Zhao X. Bai X. Y. Niu L. Song M. Q. Sun Y. G. (2012). Anti-PRRSV effect and mechanism of sodium tanshinone IIA sulfonate in vitro. *J. Asian. Nat. Prod. Res.* 14 721–728. 10.1080/10286020.2012.685727 22575045

[B140] Sun P. Sun N. Wang Z. Guo J. He Y. Li H. (2018). Matrine inhibits replication of porcine reproductive and respiratory syndrome virus (PRRSV) by influencing the activation of Nsp9. *Pak. Vet. J.* 38 359–364. 10.29261/pakvetj/2018.079

[B141] Sun P. P. Sun N. Yin W. Sun Y. G. Fan K. H. Guo J. H. (2019). Matrine inhibits IL-1 beta secretion in primary porcine alveolar macrophages through the MyD88/NF-kappa B pathway and NLRP3 inflammasome. *Vet. Res.* 50:14. 10.1186/s13567-019-0671-x 31300043 PMC6626430

[B142] Thanawongnuwech R. Amonsin A. Tatsanakit A. Damrongwatanapokin S. (2004). Genetics and geographical variation of porcine reproductive and respiratory syndrome virus (PRRSV) in Thailand. *Vet. Microbiol.* 101 9–21. 10.1016/j.vetmic.2004.03.005 15201029

[B143] Tomei L. Altamura S. Bartholomew L. Biroccio A. Ceccacci A. Pacini L. (2003). Mechanism of action and antiviral activity of benzimidazole-based allosteric inhibitors of the hepatitis C Virus RNA-dependent RNA polymerase. *J. Virol.* 77 13225–13231. 10.1128/JVI.77.24.13225-13231.2003 14645579 PMC296079

[B144] Trugo L. C. (2003). “Analysis of coffee products,” in *Encyclopedia of Food Sciences and Nutrition*, 2nd Edn, eds Trugo L. C. Finglas P. M. (London: Elsevier Science), 1498–1506.

[B145] Tu Y. (2011). The discovery of artemisinin (qinghaosu) and gifts from Chinese medicine. *Nat. Med.* 17 1217–1220. 10.1038/nm.2471 21989013

[B146] Vachon M.-L. Dieterich D. (2011). The era of direct-acting antivirals has begun: the beginning of the end for HCV? *Semin. Liver Dis.* 31 399–409. 22189979 10.1055/s-0031-1297928

[B147] van Aken D. Zevenhoven-Dobbe J. Gorbalenya A. E. Snijder E. J. (2006). Proteolytic maturation of replicase polyprotein pp1a by the nsp4 main proteinase is essential for equine arteritis virus replication and includes internal cleavage of nsp7. *J. Gen. Virol.* 87(Pt 12), 3473–3482. 10.1099/vir.0.82269-0 17098961

[B148] Van Gorp H. Van Breedam W. Van Doorsselaere J. Delputte P. L. Nauwynck H. J. (2010). Identification of the CD163 protein domains involved in infection of the porcine reproductive and respiratory syndrome virus. *J. Virol.* 84 3101–3105. 10.1128/JVI.02093-09 20032174 PMC2826032

[B149] Van Hemert M. J. Snijder E. J. (2008). “The arterivirus replicase,” in *Nidoviruses*, eds Perlman S. Gallagher T. Snijder E. J. (Washington, DC: ASM Press), 433.

[B150] Van Reeth K. Labarque G. Nauwynck H. Pensaert M. (1999). Differential production of proinflammatory cytokines in the pig lung during different respiratory virus infections: correlations with pathogenicity. *Res. Vet. Sci.* 67 47–52. 10.1053/rvsc.1998.0277 10425240 PMC7126504

[B151] Van Reeth K. Van Gucht S. Pensaert M. (2002). In vivo studies on cytokine involvement during acute viral respiratory disease of swine: troublesome but rewarding. *Vet. Immunol. Immunopathol.* 87 161–168. 10.1016/s0165-2427(02)00047-8 12072230 PMC7119797

[B152] Velthuis A. J. W. T. van den Worm S. H. E. Sims A. C. Baric R. S. Snijder E. J. van Hemert M. J. (2010). Zn2+ inhibits coronavirus and arterivirus rna polymerase activity in vitro and zinc ionophores block the replication of these viruses in cell culture. *PLoS Pathog.* 6:1176. 10.1371/journal.ppat.1001176 21079686 PMC2973827

[B153] Venkatesan N. (1998). Curcumin attenuation of acute adriamycin myocardial toxicity in rats. *Br. J. Pharmacol.* 124 425–427. 10.1038/sj.bjp.0701877 9647462 PMC1565424

[B154] Venkatesan N. Punithavathi D. Arumugam V. (2000). Curcumin prevents adriamycin nephrotoxicity in rats. *Br. J. Pharmacol.* 129 231–234. 10.1038/sj.bjp.0703067 10694226 PMC1571849

[B155] Vo T.-S. Kim S.-K. (2010). Potential Anti-HIV agents from marine resources: an overview. *Mar. Drugs* 8 2871–2892. 10.3390/md8122871 21339954 PMC3039460

[B156] Wang H. M. Liu T. X. Wang T. Y. Wang G. Liu Y. G. Liu S. G. (2018). Isobavachalcone inhibits post-entry stages of the porcine reproductive and respiratory syndrome virus lifecycle. *Arch. Virol.* 163 1263–1270. 29411137 10.1007/s00705-018-3755-4 PMC7086980

[B157] Wang M. Ng K. K. Cherney M. M. Chan L. Yannopoulos C. G. Bedard J. (2003). Non-nucleoside analogue inhibitors bind to an allosteric site on HCV NS5B polymerase. Crystal structures and mechanism of inhibition. *J. Biol Chem.* 278 9489–9495. 10.1074/jbc.M209397200 12509436

[B158] Wang R. Nan Y. Yu Y. Yang Z. Zhang Y. J. (2013a). Variable interference with interferon signal transduction by different strains of porcine reproductive and respiratory syndrome virus. *Vet. Microbiol.* 166 493–503. 10.1016/j.vetmic.2013.07.022 23953026

[B159] Wang R. Nan Y. Yu Y. Zhang Y. J. (2013b). Porcine reproductive and respiratory syndrome virus Nsp1β inhibits interferon-activated JAK/STAT signal transduction by inducing karyopherin-α1 degradation. *J. Virol.* 87 5219–5228. 10.1128/JVI.02643-12 23449802 PMC3624296

[B160] Wang R. Xiao Y. Opriessnig T. Ding Y. Yu Y. Nan Y. (2013c). Enhancing neutralizing antibody production by an interferon-inducing porcine reproductive and respiratory syndrome virus strain. *Vaccine* 31 5537–5543. 10.1016/j.vaccine.2013.09.023 24063978

[B161] Wang R. Zhang Y. J. (2014). Antagonizing interferon-mediated immune response by porcine reproductive and respiratory syndrome virus. *Biomed. Res Int.* 2014:315470. 10.1155/2014/315470 25101271 PMC4101967

[B162] Wang X. B. Cui B. A. Wei Z. Y. Qiu J. L. Duan-Hong X. U. (2008). Studies on antiviral effect of chlorogenic acid on PRRSV in vitro. *J. Agric. Sci. Technol.* 10 107–110.

[B163] Wang Z.-W. Sun N. Wu C.-H. Jiang J.-B. Bai Y.-S. Li H.-Q. (2013). In vitro antiviral activity and underlying molecular mechanisms of *Dipotassium glycyrrhetate* against porcine reproductive and respiratory syndrome virus. *Antiviral Ther.* 2013 997–1004. 10.3851/IMP2662 23872789

[B164] Wissink E. H. J. Kroese M. V. Wijk H. A. R. V. Rijsewijk F. A. M. Rottier P. J. M. (2005). Envelope protein requirements for the assembly of infectious virions of porcine reproductive and respiratory syndrome virus. *J. Virol.* 79 12495–12506. 16160177 10.1128/JVI.79.19.12495-12506.2005 PMC1211556

[B165] Wolkerstorfer A. Kurz H. Bachhofner N. Szolar O. H. J. (2009). Glycyrrhizin inhibits influenza A virus uptake into the cell. *Antiviral Res.* 83 171–178. 19416738 10.1016/j.antiviral.2009.04.012 PMC7126985

[B166] Wongyanin P. Buranapraditkul S. Yoo D. Thanawongnuwech R. Roth J. A. Suradhat S. (2012). Role of porcine reproductive and respiratory syndrome virus nucleocapsid protein in induction of interleukin-10 and regulatory T-lymphocytes (Treg). *J. Gen. Virol.* 93(Pt 6), 1236–1246. 10.1099/vir.0.040287-0 22422061

[B167] Wootton S. K. Rowland R. R. Yoo D. (2002). Phosphorylation of the porcine reproductive and respiratory syndrome virus nucleocapsid protein. *J. Virol.* 76 10569–10576. 12239338 10.1128/JVI.76.20.10569-10576.2002 PMC136587

[B168] Wootton S. K. Yoo D. (2003). Homo-oligomerization of the porcine reproductive and respiratory syndrome virus nucleocapsid protein and the role of disulfide linkages. *J. Virol.* 77 4546–4557. 10.1128/jvi.77.8.4546-4557.2003 12663761 PMC152152

[B169] Wu C. Xu Z. Gai R. Huang K. (2016). Matrine ameliorates spontaneously developed colitis in interleukin-10-deficient mice. *Int. Immunopharmacol.* 36 256–262. 10.1016/j.intimp.2016.04.038 27179305

[B170] Xie J. Zhou H. Cui J. Chen Y. Zhang M. Deng S. (2014). Inhibition of porcine reproductive and respiratory syndrome virus by specific siRNA targeting Nsp9 gene. *Infect. Genet. Evol.* 28 64–70. 10.1016/j.meegid.2014.08.008 25149224

[B171] Xie Y. Sun H.-X. Li D. (2009). Platycodin D is a potent adjuvant of specific cellular and humoral immune responses against recombinant hepatitis B antigen. *Vaccine* 27 757–764. 10.1016/j.vaccine.2008.11.029 19041358

[B172] Xie Y. Ye Y. P. Sun H. X. Li D. (2008). Contribution of the glycidic moieties to the haemolytic and adjuvant activity of platycodigenin-type saponins from the root of *Platycodon grandiflorum*. *Vaccine* 26 3452–3460. 10.1016/j.vaccine.2008.04.023 18501482

[B173] Xu Y. Li S. Chen R. Li G. Barish P. A. You W. (2010). Antidepressant-like effect of low molecular proanthocyanidin in mice: Involvement of monoaminergic system. *Pharmacol. Biochem. Behav.* 94 447–453. 10.1016/j.pbb.2009.10.007 19857512

[B174] Yang M. Lu Y. Ma Y. Wu G. Beier R. C. Hou X. (2015). Inhibition of porcine reproductive and respiratory syndrome virus in vitro by forsythoside A. *Intern. J. Pharmacol.* 11 394–399. 10.3923/ijp.2015.394.399

[B175] Yang Q. Gao L. Si J. Sun Y. Liu J. Cao L. (2013). Inhibition of porcine reproductive and respiratory syndrome virus replication by flavaspidic acid AB. *Antiviral Res.* 97 66–73. 10.1016/j.antiviral.2012.11.004 23178515

[B176] Yang R. Yuan B. Ma Y. Zhou S. (2017). The anti-inflammatory activity of licorice, a widely used Chinese herb. *Pharm. Biol.* 55 5–18. 10.1080/13880209.2016.1225775 27650551 PMC7012004

[B177] Yang Y. Gorzelanny C. Bauer A. T. Halter N. Komljenovic D. Buerle T. (2015). Nuclear heparanase-1 activity suppresses melanoma progression via its DNA-binding affinity. *Oncogene* 34 5832–5842. 10.1038/onc.2015.40 25745999

[B178] Yin W. Mao C. Luan X. Shen D.-D. Shen Q. Su H. (2020). Structural basis for inhibition of the RNA-dependent RNA polymerase from SARS-CoV-2 by remdesivir. *Science* 368:eabc1560. 10.1126/science.abc1560 32358203 PMC7199908

[B179] Yong J. Wu X. Lu C. (2015). Anticancer advances of matrine and its derivatives. *Curr. Pharm. Des* 21 3673–3680. 10.2174/1381612821666150122123748 25613788

[B180] Zhang A. Sun H. Qiu S. Wang X. (2013). Advancing drug discovery and development from active constituents of yinchenhao tang, a famous traditional chinese medicine formula. *Evid. Based Complement. Altern. Med.* 2013:257909. 10.1155/2013/257909 24191164 PMC3804150

[B181] Zhang M. Du T. Long F. Yang X. Sun Y. Duan M. (2018a). Platycodin D Suppresses Type 2 porcine reproductive and respiratory syndrome virus. In primary and established cell lines. *Viruses Basel* 10:e010657. 10.3390/v10110657 30469357 PMC6266211

[B182] Zhang M. Wu Q. Chen Y. Duan M. Tian G. Deng X. (2018b). Inhibition of proanthocyanidin A2 on porcine reproductive and respiratory syndrome virus replication in vitro. *PLoS One* 13:e0193309. 10.1371/journal.pone.0193309 29489892 PMC5831109

[B183] Zhang Q. Xue-kun G. Li G. Chen H. Ning L. Xiaojuan J. (2014). MicroRNA-23 inhibits PRRSV replication by directly targeting PRRSV RNA and possibly by upregulating type I interferons. *Virology* 450–451 182–195. 10.1016/j.virol.2013.12.020 24503081

[B184] Zhang S.-L. Wu Y.-C. Cheng F. Guo Z.-Y. Chen J.-F. (2016). Anti-PRRSV effect and mechanism of tetrahydroaltersolanol C in vitro. *J. Asian. Nat. Prod. Res.* 18 303–314. 10.1080/10286020.2015.1072516 26488075

[B185] Zhao S. Ge X. Wang X. Liu A. Guo X. Zhou L. (2015). The DEAD-box RNA helicase 5 positively regulates the replication of porcine reproductive and respiratory syndrome virus by interacting with viral Nsp9 in vitro. *Biophys. J.* 195 217–224. 10.1016/j.virusres.2014.10.021 PMC7114378 25449571

[B186] Zhou E. M. Clavijo A. Jiang Z. Ameri-Mahabadi M. Zimmerman J. J. (2004). Induction of auto-anti-idiotypic antibodies specific for antibodies to matrix and envelope glycoprotein from pigs experimentally infected with porcine reproductive and respiratory syndrome virus. *Vet. Immunol. Immunopathol.* 101 49–59. 10.1016/j.vetimm.2004.03.007 15261692

[B187] Zhou Y. Zheng H. H. Gao F. Tian D. B. Yuan S. (2011). Mutational analysis of the SDD sequence motif of a PRRSV RNA-dependent RNA polymerase. *Sci. China Life Sci.* 54 870–879. 10.1007/s11427-011-4216-4 21922433 PMC7088696

[B188] Ziebuhr J. Snijder E. J. Gorbalenya A. E. (2000). Virus-encoded proteinases and proteolytic processing in the Nidovirales. *J. Gen. Virol.* 81(Pt 4), 853–879. 10.1099/0022-1317-81-4-853 10725411

[B189] Zuckermann F. A. Garcia E. A. Luque I. D. Christopher-Hennings J. Doster A. Brito M. (2007). Assessment of the efficacy of commercial porcine reproductive and respiratory syndrome virus (PRRSV) vaccines based on measurement of serologic response, frequency of gamma-IFN-producing cells and virological parameters of protection upon challenge. *Vet. Microbiol.* 123 69–85. 10.1016/j.vetmic.2007.02.009 17376612

